# Brain activity patterns reflecting security perceptions of female cyclists in virtual reality experiments

**DOI:** 10.1038/s41598-024-81271-8

**Published:** 2025-01-04

**Authors:** Mohammad Arbabpour Bidgoli, Arian Behmanesh, Navid Khademi, Phromphat Thansirichaisree, Zuduo Zheng, Sara Saberi Moghadam Tehrani, Sajjad Mazloum, Sirisilp Kongsilp

**Affiliations:** 1 School of Civil Engineering, College of Engineering, University of Tehran, Tehran, Iran; 2 Thammasat School of Engineering, Faculty of Engineering, Thammasat University Rangsit, Klong Luang, Pathumthani Thailand; 3 School of Civil Engineering, Faculty of Engineering, Architecture and Information Technology, The University of Queensland, Brisbane, Australia; 4 Department of Physics, KTH Royal Institute of Technology, Stockholm, Sweden; 5 Department of Computer Engineering, Faculty of Engineering, Kasetsart University, Bangkok, Thailand

**Keywords:** Cognitive neuroscience, Civil engineering, Computer science

## Abstract

Active transportation, such as cycling, improves mobility and general health. However, statistics reveal that in low- and middle-income countries, male and female cycling participation rates differ significantly. Existing literature highlights that women’s willingness to use bicycles is significantly influenced by their perception of security. This study employs virtual reality (VR) cycling simulation and electroencephalography (EEG) analysis to investigate factors influencing female cyclists’ perceptions of security in Tehran. A total of 52 female participants took part in four scenarios within a VR bicycle simulator, which simulates various environmental settings. In this experiment, participants’ brainwave signals are gathered through an EEG device, and a questionnaire with their stated preferences is filled out. The Gaussian mixture approach is used to cluster brainwave patterns based on security perception from EEG data. Subsequently, four supervised machine learning methods, random forest, support vector machine, logistic regression, and multilayer perceptron, are utilized to classify influential factors on security perception using clustered EEG data. Consequently, the support vector machine model, with an F1 score of 0.74, appears to be the most effective technique for the classification of environmental and surveillance factors. Furthermore, the SelectKBest algorithm determines that factors such as the presence of obstacles like kiosks, cycling routes passing through tunnels and underpasses, the level of incivility in the urban cycling environment, and the presence of informal surveillance have the biggest impact on female cyclists’ security perception.

**Supplementary Information:**

The online version contains supplementary material available at 10.1038/s41598-024-81271-8.

## Introduction

Cycling has recently gotten a lot of attention as a potential way to improve public health and alleviate traffic in urban areas. Global endeavors have been undertaken to enhance bicycle infrastructure, encompassing initiatives such as establishing dedicated cycling lanes and implementing shared bicycle systems. Existing literature emphasizes that women’s tendency to use bicycles as a mode of transportation is notably impacted by their perception of security^1^. Whenever women feel insecure or think that potential for criminal incidents exists while traveling, they tend to change their travel plans more than men do^2^. People who use active transportation means such as cycling spend more time in urban areas than those who use other modes of transportation; thus, they may be more vulnerable to security challenges. Furthermore, cycling exposes women to a broader range of their immediate surroundings; thus, it is critical to adequately address security-related concerns to promote women’s cycling participation.

Previous studies^3–9^ have investigated the significance of personal security concerns and the fear of sexual harassment in women’s transportation patterns. However, there is a knowledge gap and an urgent need to conduct research on the influential factors and their varying degrees of impact on women’s perceptions of security, with a particular focus on female cyclists. This has motivated the current study to investigate this issue by considering Tehran, the capital of Iran, as a case study. Despite constituting half of the potential user demographic for the municipal bicycle system in Tehran, empirical data reveals that a mere approximately 5% of daily urban cycling trips are undertaken by women^10^. While existing research provides valuable insights into the general nature of this phenomenon, it is crucial to acknowledge the unique challenges presented by Tehran’s specific cultural, religious, and legal landscape. A dedicated and comprehensive research effort seems necessary to gain a deeper understanding of this issue within this context.

Thus, using data collected from Tehran, this study aims to understand the factors that influence the perception of cycling security among female cyclists in an urban area. Examining this issue and identifying the factors that shape women’s perspectives on security while participating in cycling activities has the potential to enable engineers to design effective practical implications. These implications can improve cycling security and create an environment suitable for higher female cycling participation.

### Quantifying perceived security with brainwave data

The current study used a neurophysiological method to analyze electroencephalography (EEG) data to investigate the perceived security of female cyclists. We were looking for subtle neurophysiological indicators that may represent alertness, caution, or stress responses, all of which can be associated with perceived security by examining patterns of brainwave activity. This method allowed us to investigate the experience of security in a more objective and scientifically rigorous manner, shedding light on the neurological basis of this complex psychological concept.

Using EEG in transportation engineering proves to be advantageous for investigating travelers’ behavior. This is due to its ability to effectively examine the electrical activity inside the brain, hence facilitating a comprehensive comprehension of cognitive processes and mental states during the trip^11^. Prior studies have identified several methodologies for utilizing EEG in analyzing transportation behavior. Cao et al.^12^ used EEG to analyze brain activity during driving, capturing cognitive effort and attention levels. Arefnezhad et al.^13^ employed EEG-based measurements to examine the effects of weariness and drowsiness on transportation behavior. Doudou et al.^14^ observed that indicators such as alpha power, theta waves, and sluggish eye movements can serve as potential markers for fatigue and sleep initiation. Zhou et al.^15^ showed that the utilization of EEG data has the potential to facilitate the development of fatigue detection systems, resting strategies, and scheduling recommendations aimed at mitigating the likelihood of accidents caused by exhaustion. Begum et al.^16^ employed EEG to assess emotional states and stress levels experienced within vehicles. They aimed to assist designers in creating transportation environments that promote well-being, comfort, and positive user experiences. Finally, following the advancement of emerging transportation technologies, such as autonomous vehicles, Shin et al.^17^ undertook a study employing EEG to explore potential enhancements in autonomous vehicle actions to avoid potential hazards by analyzing human brainwave patterns.

The literature review indicates a growing preference for using EEG in the field of transportation research and travel behavior analyses. The current study investigates the association between female cyclists’ brainwaves and their perception of security using EEG. To the best of our knowledge, this investigation has not been previously conducted.

### Application of machine learning algorithms in cognitive studies

In our pursuit of a deeper understanding of the factors influencing the perceived security of female cyclists, we have employed a machine learning model to explore the multifaceted relationship between perceived security and a set of independent variables.

Machine learning has the potential to reveal the underlying rational mechanism in human behavior^18^. Various studies have employed machine learning in travel behavior analyses. In a study conducted by Omrani^19^, the transportation mode choice of passengers in Luxembourg was examined as a function of individual characteristics, place of residence, workplace, and characteristics of various modes of transportation. They employed four machine learning tools, namely support vector machine (SVM), multinomial logistic regression (MNL), artificial neural network (ANN) with multilayer perceptron (MPL), and ANN with radial basis function (RBF). Tang et al.^20^ have utilized learning algorithms for the purpose of forecasting and simulating transportation demand. They employed deep neural network (DNN) techniques and smart-card information to compute the hourly demand of bus passengers. Yang et al.^21^ conducted a study examining drivers’ behavior at unsignalized intersections. They employed a random forest (RF) approach and collected data from professional drivers participating in test driving sessions within a real-time driving simulator. Their study aimed to assess the magnitude of the impact of distinct behavioral features of drivers on the decision-making process at intersections. Le et al.^22^ utilized tree-based machine learning algorithms to analyze the responses of female cyclists in the United States and Canada to identify the key characteristics of women cycling in North America. Furthermore, Kim^23^ conducted a study utilizing machine learning techniques, including RF, ANN, and extreme gradient boosting (XGB) algorithms, to analyze the mode choice behavior of commuters.

The existing body of literature also contains numerous instances of EEG data analysis and classification using machine learning techniques. Zhou et al.^15^ employed machine learning algorithms, specifically logistic regression (LR), linear discriminant analysis (LDA), adaboost (ADB), k-nearest neighbor (KNN), decision tree (DT), gradient boosting machine (GBM), and SVM, on EEG data, to identify the optimal combination of features that contribute to traffic accidents resulting from driver weariness. Balam et al.^24^ employed a novel deep learning approach utilizing a convolutional neural network (CNN) and the analysis of EEG signals to develop an intelligent and automated system for alerting drivers about drowsiness, which could effectively mitigate accidents resulting from driver fatigue. Hu et al.^25^ employed SVM to detect drivers’ exhaustion by analyzing EEG data. Similarly, Wang et al.^26^ proposed a methodology for classifying drivers’ weariness based on the analysis of brain wave patterns based on KNN, DT, SVM, and RF algorithms. Zink et al.^27^ investigated the potential influence of the surrounding environment on people’s cognitive performance while cycling using a three-class oddball hearing test. They employed two machine learning techniques, namely regularized linear discriminant analysis (rLDA) and SVM, for classification based on EEG data recorded by a mobile EEG amplifier.

According to the literature and the aim of our research, we used EEG data analysis and four machine learning classification algorithms, namely, RF, SVM, LR, and MLP in the current study to find out the factors influencing women’s perception of security while cycling.

### Investigating the building blocks of cyclists’ perceived security

Individual perspectives on cycling security, which may differ from those regarding national security or system-wide security, are discussed in this study. Security perception relates to how a cyclist gathers, interprets, and consciously experiences sensory data concerning protection and security. This perception may differ depending on the users’ location and time, demographic and socio-psychological characteristics, and system attributes. Individuals’ perception of security along cycling paths can bring them confidence in performing the cycling task and impact their desire to cycle. Consequently, researchers proposed methodologies for quantifying and measuring perceived security.

Due to the similarities between the factors influencing cyclists’ and pedestrians’ perception of security, existing literature is examined on both. Based on the previous studies and our engineering judgment, factors affecting women’s security perceptions during walking and cycling can be segmented into five distinct categories (to view the appearance of each factor in the designed virtual reality environment, see Supplementary Fig. S1 online):

- Obstacles, vegetation, and underpasses. Improved visibility in urban areas has been found to increase women’s sense of security and decrease their fear of crime^28^. Rearranging parks and removing visual barriers are two examples of improving one’s perception of security^29^ by enhancing the visual span. Women may feel insecure in green areas like parks if they do not have ample visibility, audibility, escape routes, and proximity to help^30^. According to the study conducted by Mahrous et al.^31^, visitors’ perception of security in parks may be affected by the density of vegetation. Anciaes and Jones^32^ analyzed the willingness of pedestrians to cross different forms of pedestrian crosswalk infrastructure and discovered that many women, especially those without firsthand experience of harassment, consider underpasses fit for street harassment and violence.

This study takes into account four levels of visual barriers, each with its own unique set of obstacles: a completely clear environment (OBS1), a path hindered with kiosks and stalls (OBS2 in Supplementary Fig. S1i), a route through a vegetative corridor (OBS3 in Supplementary Fig. S1ii), and bike lanes passing through an underpass or tunnel (OBS4 in Supplementary Fig. S1iii).

b. Evidence of incivility. Previous studies^29,31,34,35^ have hypothesized that indicators of incivility, including litter, abandoned vehicles, infrastructure deterioration, graffiti, and vandalism, contribute to diminished perceptions of security and elevated fear of crime in urban environments. According to the “broken windows theory "^36^, crime rates tend to rise in rundown metropolitan areas. Foster et al.^37^ found an association between the fear of crime among pedestrians and environmental neighborhood elements such as vacant houses or blocks. In addition, in urban areas, rampant signs of incivility can make women feel unsafe^35,38^.

The current study considers three different urban settings. The first level (INC1 in Supplementary Fig. S1iv) is a clean and pleasant urban setting. In the second level, buildings (INC2 in Supplementary Fig. S1v) are more subdued, with a more or less regular façade and some degradation. In Level 3 (INC3 in Supplementary Fig. S1vi), the hideous urban design, damaged buildings, shattered glass, unpleasant graffiti, hazardous bike lanes, and uneven and cracked pavement are evident.

c. Informal surveillance (presence of others). Enhancing perceived security in urban situations requires creating locations that welcome more activities and individuals. The concept of “eyes on the street” provided by Jacobs^39^ and “opportunities for surveillance” proposed by Newman^40^ suggested that the presence of people functions as informal surveillance and can cause lower crime and boost security perception. Hillier’s^41^ “theory of virtual community” further highlights that various groups of individuals’ organic movement contribute to constant observation and increase security perception^35^. The chance of being spotted by pedestrians, car traffic, or bikers can dissuade possible perpetrators^42^. Various levels of crowdedness are investigated in this paper, ranging from unoccupied to rush hour conditions.

This study considers multiple levels of occupancy. The empty state (POP1 in Supplementary Fig. S1vii) shows minimum activity with no bikes, no pedestrians, and vacant roads. The rush hour state (POP4 in Supplementary Fig. S1viii) depicts packed streets, lively sidewalks with considerable pedestrian traffic, and occupied bike lanes. Intermediate levels of congestion are also addressed for (POP2 and POP3), with varied volumes of car traffic, pedestrians, and bikes.

d. Time of day and street lighting. The time of day strongly affects visibility and perceived security, with lower perceived security reported at night, particularly among women^43^. Women may avoid commuting or using specific transportation modes at midnight due to security concerns^2,7^. Darkness has a crucial impact on women’s perception of security, driving them to avoid physical activities and active transportation modes at night^35^. Enhancing street illumination has been found to boost women’s impression of security^29^. Adequate street illumination promotes visibility, cycling control, and the belief that others can see and provide aid if needed^43^. Studies have demonstrated that good nighttime illumination greatly reduces crime rates, as darkness provides shelter for offenders^42,44^.

Foster et al.^37^ have identified inadequate ambient illumination and consequently limited informal surveillance as principal determinants that exert an influence upon the broader concept of security. In the same line, Börjesson^45^ categorized urban environments based on visibility and the ability to escape in the event of danger.

Our virtual reality (VR) simulation environments cover three levels of ambient lighting: daylight (LIG1), appropriate night lighting (LIG2 in Supplementary Fig. S1ix), and insufficient or poor night lighting (LIG3 in Supplementary Fig. S1x).

e. Formal surveillance (presence of police). Formal surveillance, such as the presence of visible or covert police officers, can mitigate harassment and impact women’s perception of security in urban areas^42^. Increasing the number of police officers and monitoring technologies in transport systems has been proven to effectively reduce emotions of anxiety among women^46,47^. This study considers two levels of surveillance: the presence of police officers (POL1 in Supplementary Fig. S1xi) or their absence (POL2).

**Fig. 1:**
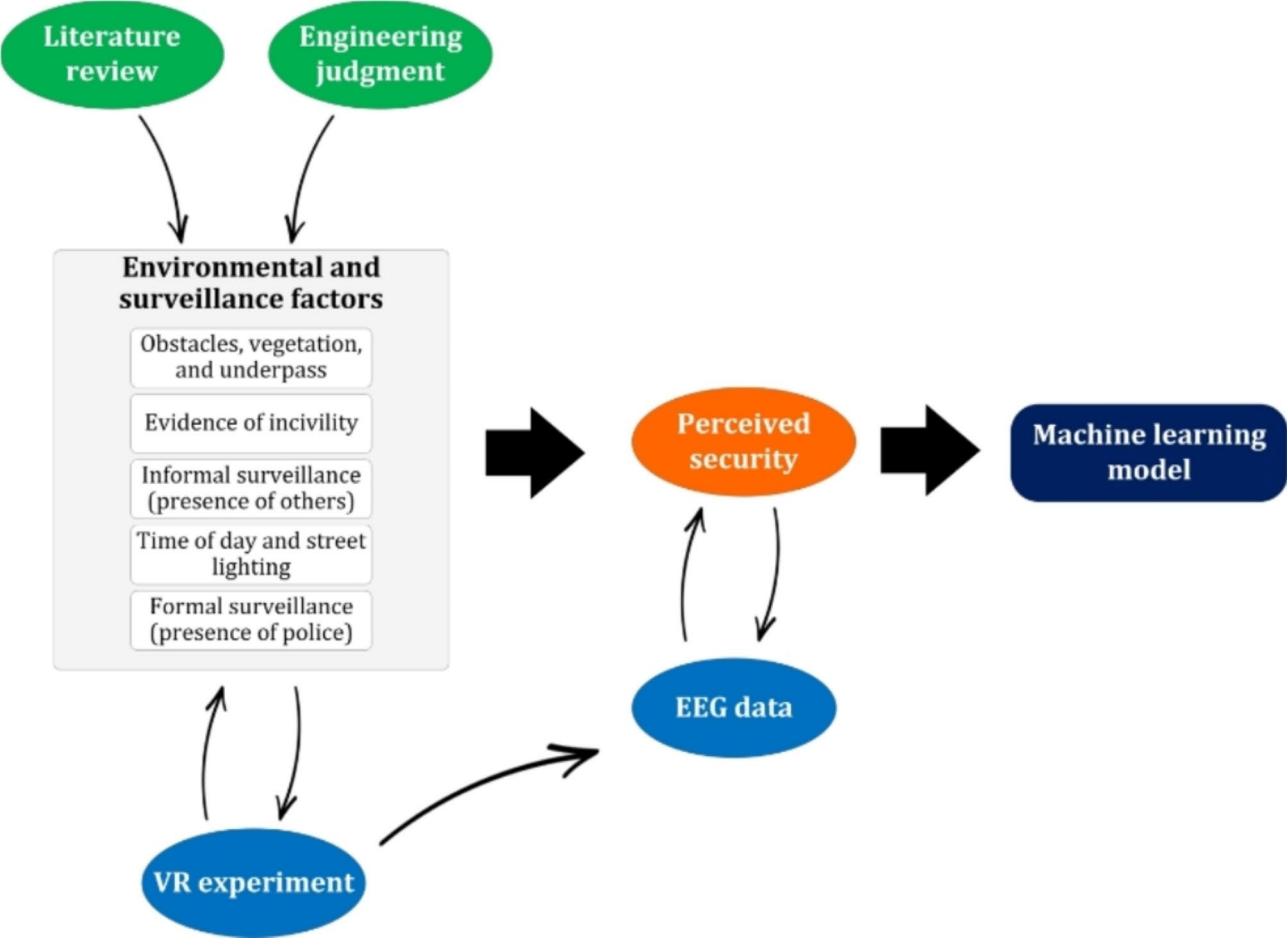
Schematic representation of the framework used in this article to analyze the female cyclists’ perceived security.

Based on the aforementioned literature, Fig. 1 depicts the framework utilized in this study to explore the factors influencing female cyclists’ perceived security.

## Experiment and data collection

This experiment builds upon previous studies^33,48^, where a VR environment was used to investigate female cyclists’ perceived security. While the VR environment and experimental setup remain consistent with earlier work, this study includes new data collection using EEG to capture brain activity during the cycling task. The data from the security perception questionnaire, previously utilized, is partially reused in this study, but the EEG data introduces an additional layer of insight unique to this research. The details of this extended experiment are provided in the following sections.

### Participants

**Fig. 2:**
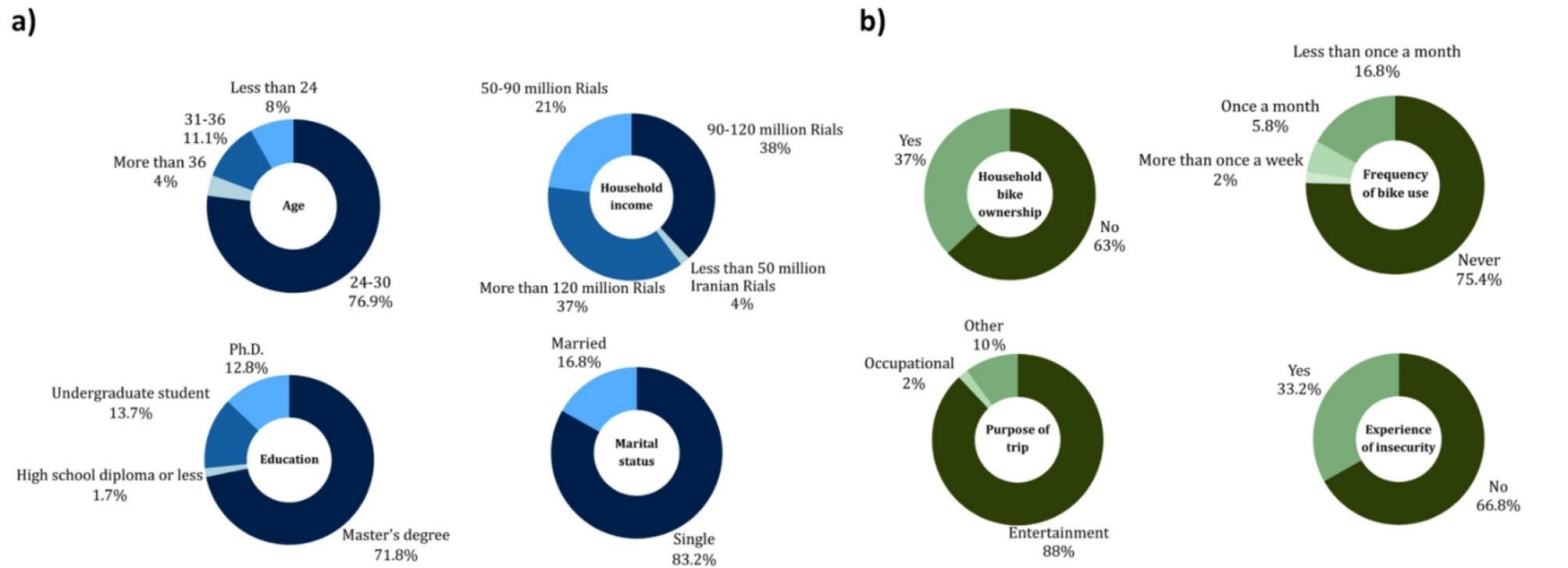
Participants’ (**a**) demographic profile and (**b**) transportation characteristics.

In this study, 52 women were recruited to participate in a VR bicycle simulator experiment. The study’s participants were selected from female cyclists residing in Tehran, with an average age of 26.8 years, ranging from 18 to 42 year, trying to have an unbiased sample. None of the participants had prior experience with a VR bicycle simulator. For detailed information regarding the current study’s participants’ demographics and transportation characteristics, refer to Fig. 2.

### Apparatus

As depicted in Supplementary Fig. S2a, a two-degree-of-freedom VR-based bicycle simulator created in Tehran University’s Laboratory of Advanced Service Robots was used in this study, which provides vertical and horizontal angle modification to the participants while cycling. The simulator comprised two electric motors with a gearbox for data transmission, a stationary bicycle, an Oculus Rift VR headset with 360-degree vision, and a computer with a Core i7-7700k CPU and an Nvidia GeForce GTX 1080 GPU. Motion sensors measured the steering angle and wheel speed to establish communication between the virtual environment and the cycling simulator. The ability to transfer data from the cycling simulator to the virtual environment was made possible using Arduino IDE software version 1.8.19.

### Simulated scenarios

This study employed a VR environment composed of 16 distinct scenarios, each designed to incorporate the factors influencing women’s perceived security while cycling. These factors were identified in the section titled ‘Investigating the Building Blocks of Cyclists’ Perceived Security.’ Supplementary Table S1 presents an overview of the experimental settings employed for each scenario in this study. The experimental scenarios were created using Unreal Engine 4. The virtual environment replicated the urban settings of Tehran and incorporated custom-designed assets explicitly developed for this research (see Supplementary Fig. S2b). The asset development process involved using software such as Adobe Photoshop, Autodesk 3ds Max, Unreal Engine, and Datasmith. The asset development workflow is shown in Supplementary Fig. S2c, and it illustrates the steps involved from conceptual design to final implementation within the virtual environment.

### Procedure

To protect participant rights, the Faculty of Psychology and Education Biomedical Research Ethics Committee at the University of Tehran approved this study (Approval ID: IR. UT. PSYEDU. REC. 1400. 009; date: 2021-05-22) and all the methods and tests performed in this research are in accordance with the relevant guidelines and regulations. All participants gave informed consent to participate in the experiment and to publish their images in the paper before initiating the test. The experiment was conducted through a series of six steps. In the first step, participants were briefed about the test and its conditions. Next, in Step 2, participants performed a warm-up test, giving them about five minutes to become used to the equipment and VR cycling simulator. In Step 3, participants engaged in the main test in which they cycled in four randomly assigned scenarios for 2 min within the VR environment. After giving the participants, 1–2 min to rest (Step 4) to prevent simulation sickness, they exited the simulator and completed a 5-point Likert scale questionnaire on perceived security designed based on prior studies^49,50^ (Step 5).

As the final step, we used the presence questionnaire developed by Witmer and Singer^51^ to gauge the extent to which participants felt present in the virtual world. Involvement, adaptation/immersion, sensory fidelity, and interface quality were explored in depth across 24 questions. Participants also reported their age, level of education, occupation, income, bicycle ownership status, car ownership status, frequency of bicycle use, and the number of experienced security mishaps. Each test took about 26 min, and the entire experiment was conducted over the course of 50 days.

## Methods

### Brainwave signals and their relationship to female cyclists’ perceptions of security

**Fig. 3:**
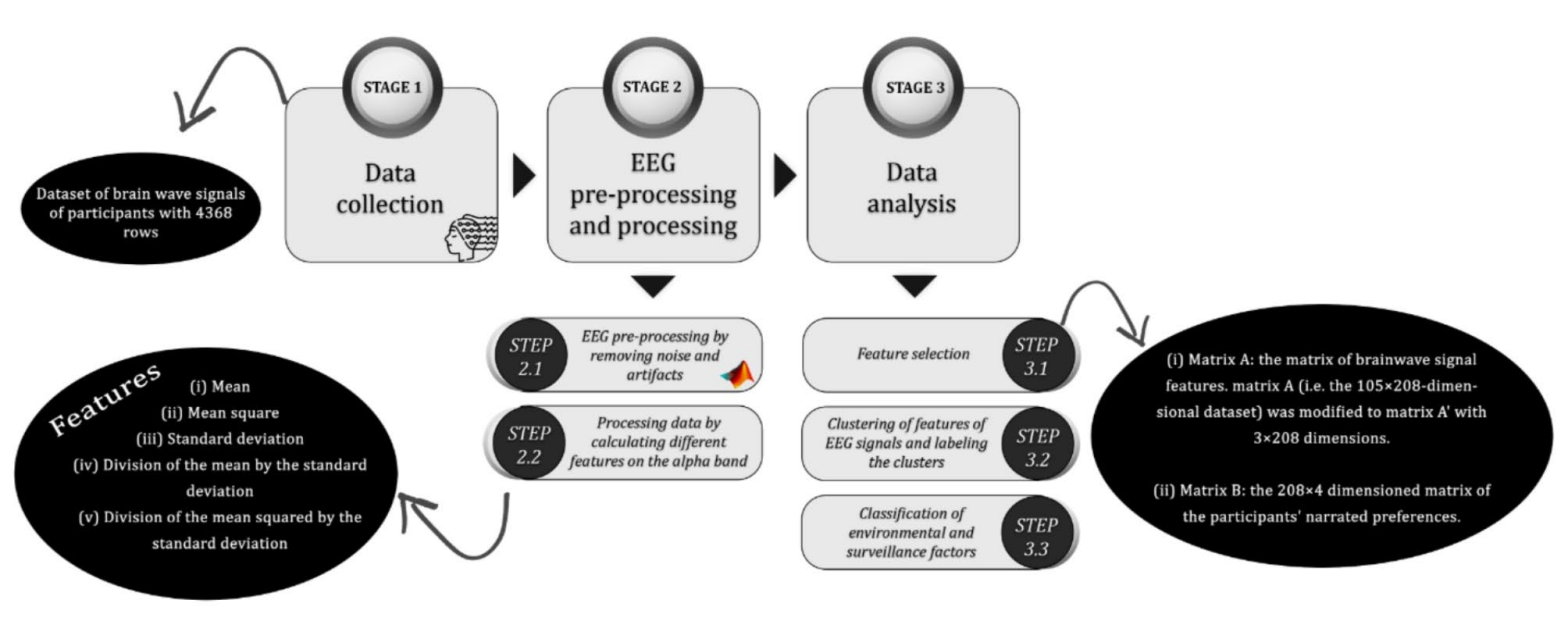
Modeling framework.

Figure 3 illustrates the methodology employed in this study, encompassing three distinct stages: Stage 1: data collection; Stage 2: pre-processing and processing of EEG data; and Stage 3: data analysis. These stages aimed to elucidate the impact of environmental and surveillance factors, identified in the ‘Investigating the Building Blocks of Cyclists’ Perceived Security’ section, on the perceived security of female cyclists. These stages will be further scrutinized in the following sections. It should be emphasized that all the methods and tests performed in this research are in accordance with the ethical guidelines and regulations for involving human participants.

### Stage 1: data collection

**Fig. 4:**
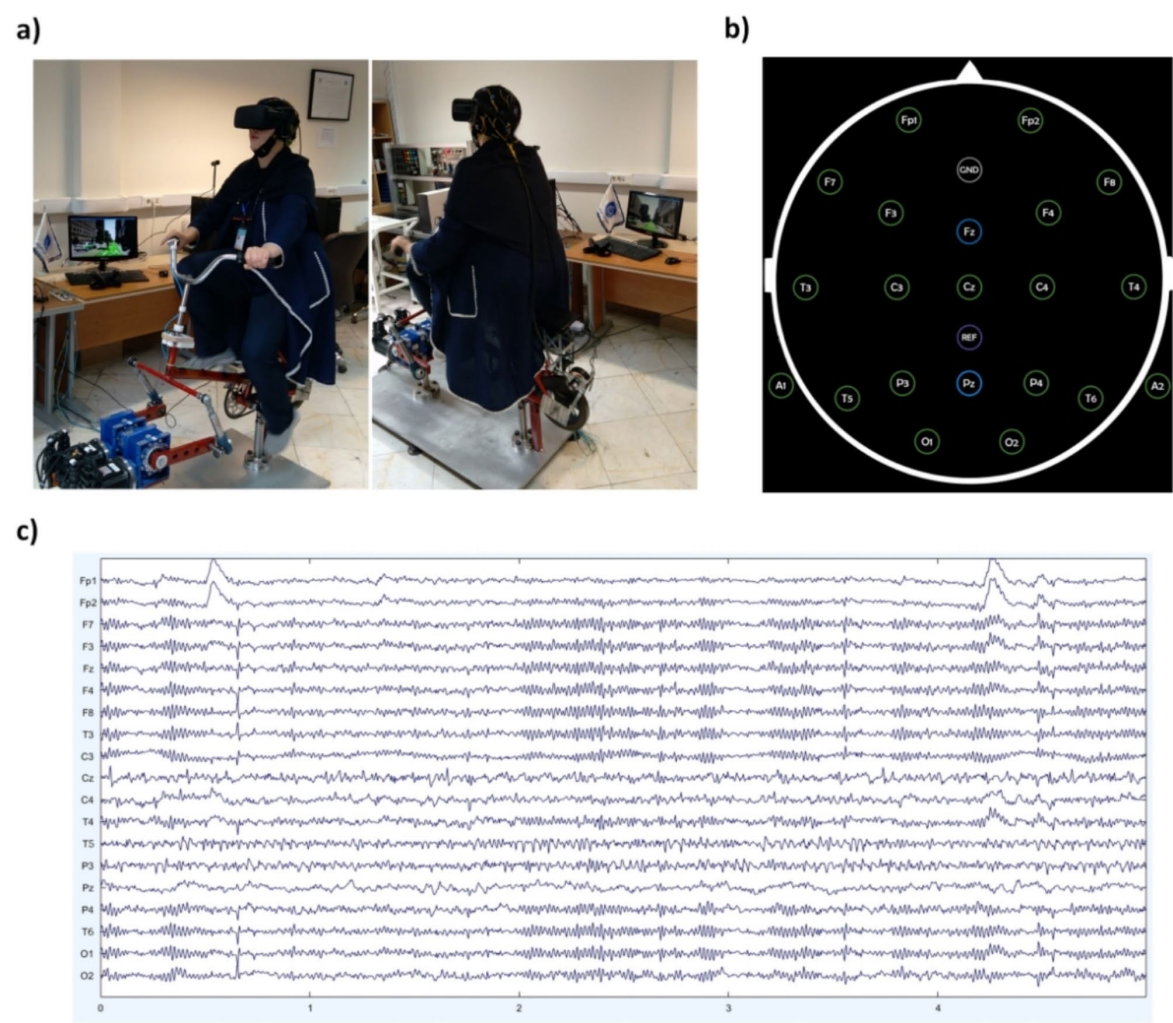
(**a**) EEG data recording of a participant riding a VR-based bicycle simulator. (**b**) Locations and labels of 21 channels of EEG device. (**c**) A sample of recorded brainwave signals.

In this study, data collection involved recording EEG data from cyclists using a VR-based bicycle simulator, as illustrated in Fig. 4.a. Brainwave signals were acquired using a 21-channel EEG device (Fig. 4.b) with a sampling rate of 256 Hz. The study recorded neurological responses from 52 female cyclists across 4 scenarios, resulting in $\:(52\times\:4)\times\:21=\text{4,368}$ rows of data for each experimental scenario. Although the testing duration varied per participant, it averaged 35 s per scenario. Given the 256 Hz sampling rate, each participant and EEG channel yielded approximately $\:35\times\:256=\text{8,960}$ data points. Consequently, the complete dataset comprises a matrix with dimensions of $\:\text{4,368}\times\:\text{8,960}$ per test. Figure 4.c exhibits the unprocessed brain signal samples for the 21 channels of the EEG device. It is evident that distinct signal patterns are observable for each channel. The alterations in these patterns have the potential to serve as a foundation for the extraction of essential aspects required for the assessment of human perceptions.

### Stage 2: EEG pre-processing and processing

#### Step 2.1: pre-processing data by removing noise and artifacts

The primary objective of data pre-processing was to clean brainwave signals by eliminating noise and artifacts, thus enhancing the signal-to-noise ratio. The signals underwent several filtration stages to remove unwanted disturbances, following the EEG data cleaning protocol described by Bogacz et al.^52^. Initially, brainwave signals were converted from the time domain to the frequency domain using the short-time Fourier transform (STFT) technique^53^. Next, a bandpass filter (BPF) with a range of 1–30 Hz was applied to retain only the desired frequency components^54^. This step eliminates frequencies outside this range, which may originate from external sources, such as the operation of electronic equipment, or internal physiological sources^54,55^. The data were then further refined to remove disturbances caused by eye blinks and excessive body movements, using a multiple source analysis technique^56–58^. The pre-processing and processing of participants’ brainwave signal data were carried out utilizing MATLAB software version 9.9 and the EEGLAB^59^ platform version 2020.

#### Step 2.2: Processing data by calculating different features on the alpha band

The primary purpose of brainwave signal processing was to extract the most meaningful brainwave signal features that can be used as cognitive parameters for understanding female cyclists’ security perceptions. According to Palva and Palva^60^ and Yeom et al.^61^, attention and alertness to environmental and external stimuli can cause significant changes in the power spectra of the alpha brainwave frequency band in humans. In the same line, Roth and Cohen^62^, Choi et al.^63^, and Chatterjee et al.^64^ have stated that the more intensive the impact of task-related environmental stimuli on humans, the more brain activity increases in the alpha frequency band. Therefore, the hypotheses of the current study focused on changes in brainwave power spectra in the alpha frequency band. EEG signals are commonly classified into five frequency bands: delta (0–4 Hz), theta (4–8 Hz), alpha (8–12 Hz), beta (12–30 Hz), and gamma (more than 30 Hz)^65,66^.

**Fig. 5:**
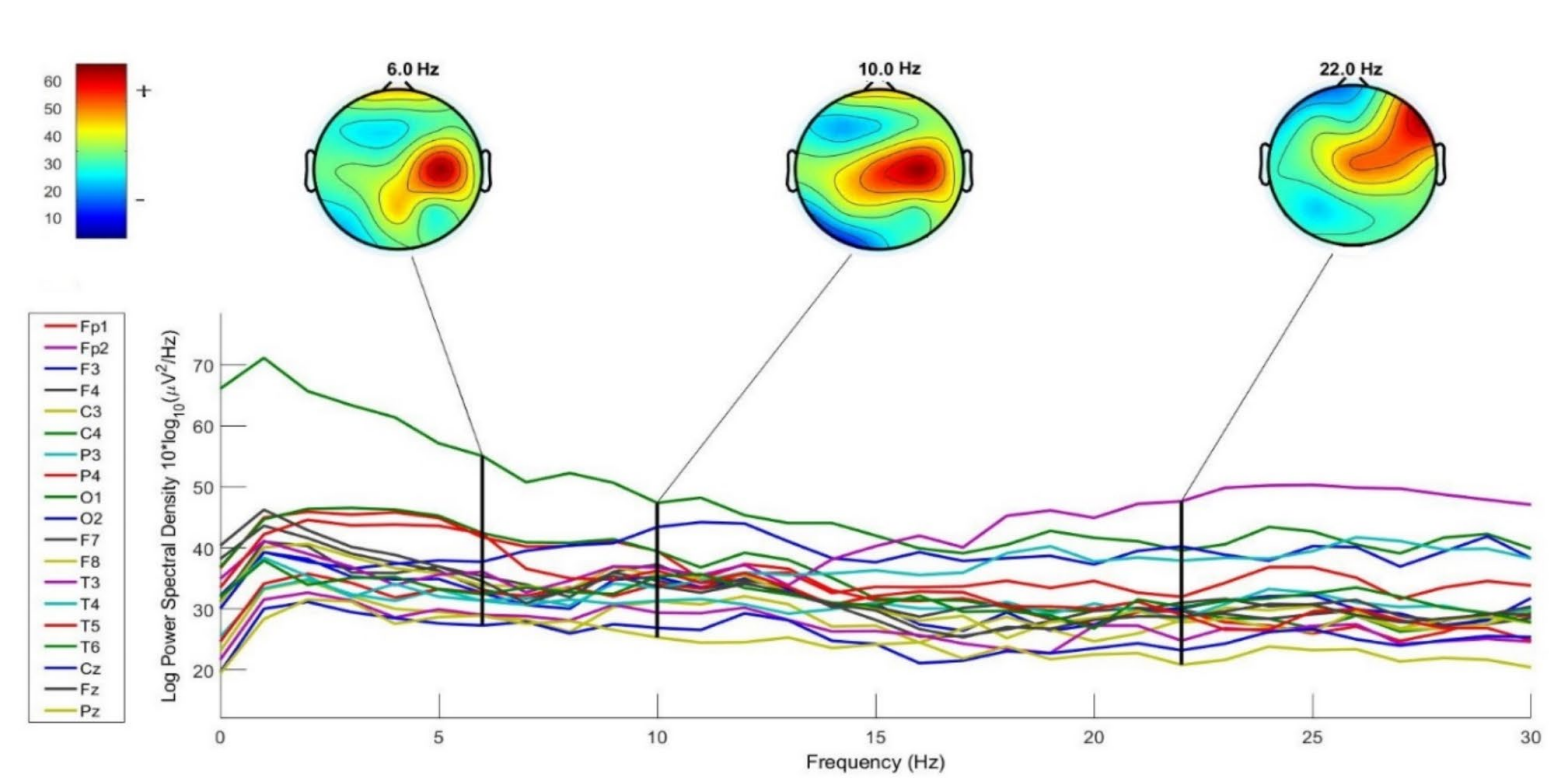
Channel spectra of EEG data of a respondent’s brainwave signals over 21 channels.

In this step, brainwave signals underwent filtration with a band pass filter method^54^, constraining the frequency range to 8–12 Hz, making alpha band signals available for analysis. Subsequently, the Welch Method^67^ was applied to compute the power spectral density (PSD) of the brainwave signals in intervals of one second for each channel’s row of data. Figure 5 depicts an example of a calculated PSD for a particular participant in logarithmic form. This figure also shows brain activity at frequencies of 6, 10, and 22 Hz, representing the theta, alpha, and beta frequency bands, respectively.

Afterward, a normalization procedure was employed, according to Quackenbush^68^ and Singh^69^. This procedure served a dual purpose: firstly, to mitigate potential errors stemming from variations in absolute brainwave signal power among participants, and secondly, to facilitate meaningful comparisons of relative brainwave signal power across diverse experimental conditions. The normalization involved adjusting all processed signal magnitudes relative to a mean of zero and a standard deviation of one.

Five features were extracted from the spectral power normalized in the alpha frequency band of 21 EEG channels for 208 rows of data:

(i) Mean,
(ii) Mean square,
(iii) The standard deviation of the spectral power of signals,
(iv) Division of the mean spectral power of signals by their standard deviation and.
(v) Division of the mean squared spectral power of signals by their standard deviation.

These were utilized as metrics for understanding the perception of security among female cyclists in the subsequent stages.

### Stage 3: data analysis

This stage aimed to classify the environmental and surveillance factors influencing the security perception of female cyclists. Examining the characteristics inherent to brainwave signals was a pivotal step in achieving this objective. This can potentially lead to (1) establishing a relationship between brainwave signals and the perception of security, (2) using machine learning techniques for clustering brainwave signals, and (3) classifying environmental and surveillance factors that influence the security perception measured by brainwave signals.

#### Step 3.1: feature selection by establishing the correlation between brainwave signals and the security perceptions of female cyclists

The initial step in confirming the study’s primary hypothesis, which posited alterations in brainwave signal power in response to perceived insecurity during cycling, involved feature selection by establishing a correlation between the extracted features of brainwave signals and the participants’ stated preferences. In this regard, we assessed the correlation between five distinct features derived from the brainwave signals (Step 2.2), obtained through 21 channels (Fig. 4.b), and the participants’ stated preferences, as measured by the questionnaire (through the PS1 to PS4 items provided in Table 1).

The process of feature selection was initiated by obtaining the following two datasets from the VR experiments:

1. Matrix A: the matrix of brainwave signal features described in the preceding stages was obtained in a $\:208\times\:105$-dimensional dataset. It contained five primary features, introduced in Step 2.2, for each of the 21 EEG channels and was evaluated on 52 female cyclists within 4 scenarios. In this dataset, each cell represents a feature quantity of the brainwave signal, such as the mean spectral power of the brainwave signal in the alpha frequency band for an EEG channel, calculated from the experience of a single participant in a single scenario (Fig. 3).
2. Matrix B: the $\:208\times\:4$ dimensioned matrix of the participants’ stated preferences corresponds to the four items of security perception (i.e., PS1 to PS4 in Table 1). Each cell in this dataset indicates each participant’s level of security perception, as measured on a 5-point Likert scale for each question.

After capturing the primary features of the brainwave signal, the analyses revealed that the mean spectral power of three channels, F4, C4, and F8 (in Fig. 4b) in the alpha frequency range had the highest Pearson correlation (refer to Table 1) with the items of the women’s security perception (PS1, PS2, PS3, and PS4). This approach can potentially reduce the dimensionality of the EEG data while focusing on the most relevant features of the research question. Therefore, the mean spectral power of the brainwave signal in the alpha frequency band for these three channels was chosen as the primary feature, and matrix A (i.e., the $\:208\times\:105$-dimensional dataset) was modified to matrix A’ with $\:208\times\:3$ dimensions. From the 5 features, one was selected, and from the 21 channels, three were chosen for the brainwave signals of the 52 female cyclists within 4 scenarios.

In accordance with the foundations of cognitive neuroscience^70^, it can be understood that channels F4, C4, and F8 (in Fig. 4b) encompass signals that capture alterations in human sensory perception. The right hemisphere of the frontal, central, and parietal lobes^70^ in which these channels are located constitutes a significant neural region associated with higher-order cognitive functions, perception, and sensory integration. These signal changes are anticipated to play a pivotal role in influencing the perception of security among female cyclists during their activities.

**Table 1:** Correlation between female security perception (measured on a 5-point likert scale) and brainwave signals in the alpha band, significant at * 0.05 level (2-tailed) and ** 0.01 level (2-tailed).

Items		F4Mean	C4Mean	F8Mean
PS1 (I feel safe while cycling on this route)	Correlation coefficient	0.407**	0.446**	0.292*
	P-value	0.002	0.001	0.029
PS2 (I do not feel worried about being robbed when using the bicycle on this cycling route)	Correlation coefficient	0.368**	0.344**	0.233
	P-value	0.005	0.009	0.085
PS3 (I do not feel worried about being abused when using the bicycle on this cycling route)	Correlation coefficient	0.385**	0.371**	0.216*
	P-value	0.003	0.005	0.011
PS4 (Generally, I feel safe using the bicycle on this cycling route)	Correlation coefficient	0.323*	0.359**	0.209*
	P-value	0.015	0.007	0.012

### Step 3.2: clustering the features of EEG signals

- Clustering procedure. Clustering can be a useful tool to identify natural groupings in the data, which can then be used to improve the performance of the modeling. In this regard, the objective was to categorize the brainwave signal features that had the most correlation with the perceived security of the participants (based on the previous step) so that we could have credible labeling for EEG data alongside environmental and surveillance factors.

First, as depicted in Fig. 3, the input matrix of brainwave signal features was matrix A’ holding a $\:208\times\:3$-dimension, which denotes one feature (i.e., mean spectral power) of brainwave signals over three channels F4, C4, and F8, and with respect to 52 participants. Then, we used a Gaussian Mixture Model (GMM) for the unsupervised categorization of EEG signal features, with the parameters of the Gaussian distributions estimated using the Expectation-Maximization (EM) algorithm. GMM was chosen for clustering EEG signal features due to its ability to model the complex, multimodal distributions often observed in neurophysiological data, such as continuous EEG signal features^71–73^. The EM algorithm, known for providing maximum likelihood estimates and strong convergence properties, was used to estimate the GMM parameters^74^. We chose to use five clusters to align with predefined security perception levels while balancing model complexity^75^, ensuring a meaningful interpretation of our results. This approach allows for probabilistic cluster assignments, capturing the inherent uncertainty in EEG signal variations, making it ideal for our analysis. Overall, this combination of model choice and parameter settings provided an efficient framework for analyzing complex patterns in our EEG data while maintaining interpretability within the context of security perceptions.

The mean spectral power of brainwave signals in the alpha frequency range for channels C4, F4, and F8 were aggregated into five clusters. Note that at this point, the clusters lacked names and numerical order. The outcomes of this step were used in the subsequent steps to investigate the relationship between environmental and surveillance factors and the perception of women’s security while cycling.

b. Clusters’ labeling. Table 1 acknowledges that the mean spectral power of brainwave signals in the alpha frequency band is correlated with the perception of the security of female cyclists. The higher the mean spectral power of brainwave signals in a cluster, the more secure the user perceives the cycling environment. Thus, labeling the five clusters of brain signals derived from Step 3.2.a can be linguistically ascribed to very secure, secure, somewhat secure, not very secure, and not secure at all.

**Fig. 6:**
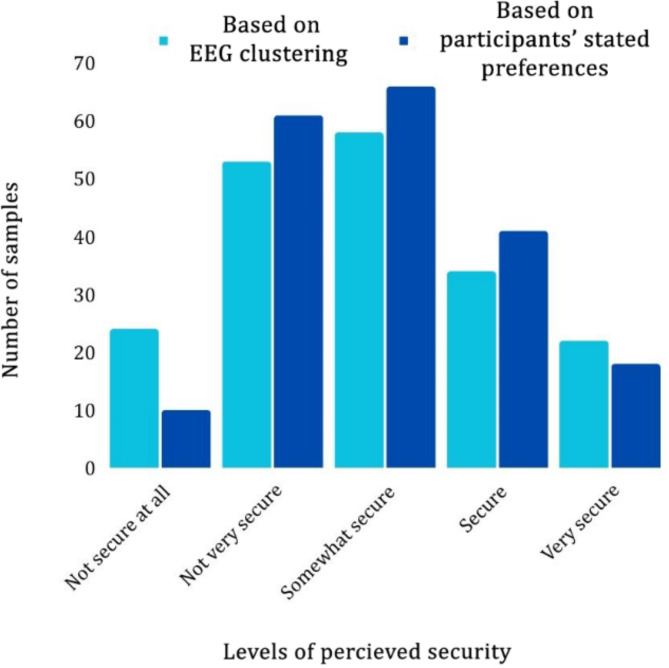
Sample distributions within the five security perception categories for EEG clustering and participants’ stated preferences.

Figure 6 compares the distinct categories of women’s perception of security as measured by EEG signals (the mean spectral power) and the participants’ stated preferences in the cycling test. As is evident, the sample distributions within the five security perception categories for both datasets are comparable. On average, 76% of EEG clusters are consistent with participants’ stated preferences at the same situational levels. This demonstrates that the brain signals of female cyclists were correctly categorized and labeled to reflect their perception of security.

### Step 3.3: classification of environmental and surveillance factors

The degree to which each environmental and surveillance factor affects the perceived security of female cyclists was assessed by classifying them into different categories based on the level of security perception deduced from brainwave signal features. This was achieved using machine learning algorithms, which will be detailed in this section.

- Classifying procedure. In this phase, the dataset was segregated into distinct categories, using environmental and surveillance parameters as model inputs and EEG signals labeled as model outputs. The Scikit-learn library in Python was utilized to implement four supervised classification methods: (i) RF, (ii) SVM, (iii) LR, and (iv) MLP. The selection of these algorithms was guided by their frequent and effective use in similar studies, as well as consultations with domain experts. Each of these algorithms could be applied with hyperparameter values (e.g., number of iterations, learning rate, and number of estimators) reported in Table 2. The GridSearch algorithm^76^ determines the optimal hyperparameter values. GridSearch is a brute-force hyperparameter optimization algorithm that systematically evaluates all possible combinations of hyperparameter values for the machine learning models. This was achieved by constructing a grid of all possible hyperparameter values and then training and evaluating the model on each combination. The combination of hyperparameter values that produced the best performance on the evaluation set was selected as the optimal hyperparameter configuration.
- K-Fold cross-validation. The K-Fold cross-validation^77^ is a widely used machine learning technique for assessing a predictive model’s performance and generalization. To prevent overfitting, improve the reliability and accuracy, and increase the efficiency of the models, especially considering the presence of data imbalance, we evaluated our modeling results using the following techniques:

- Data augmentation: We up-sampled the training dataset up to five times, using 80% of the data for training and 20% for testing. This helped to reduce the overfitting of the models to the training data and improve their performance on unseen data.
- Cross-validation: We used cross-validation to calculate the average accuracy of the models on the training and testing sets. This helped to provide a more reliable estimate of the models’ generalization performances.

**Table 2:** Hyperparameters of the EEG clustering and environmental and surveillance factors classification.

Method	Hyper parameters
Clustering	
GMM	Covariance_type=’diag’, init_params=’kmeans’, max_iter = 200, n_components = 5, n_init = 1, tol = 0.001, verbose = 0, verbose_interval = 10
Classifying	
RF	Bootstrap = True, ccp_alpha = 0.0, criterion=’gini’, min_samples_leaf = 1, min_samples_split = 2, n_estimators: 1200
SVM	C = 1.0, kernel=’rbf’, degree = 3, gamma=’scale’, coef0 = 0.0, shrinking = True, probability = False, tol = 0.001, max_ter=-1
LR	Penalty=’l2’, dual = False, tol = 0.0001, C = 1.0, fit_intercept = True, solver=’lbfgs’, max_iter = 100, multi_class=’auto’
MLP	Hidden_layer_sizes = 200, activation=’relu’, solver=’adam’, alpha = 0.0001, learning_rate_init = 0.001, max_iter = 200, momentum = 0.9, early_stopping = True

To ascertain the efficacy of the employed models, two widely accepted validation criteria were employed^78^:

1. Accuracy: The primary performance metric for classifiers is the proportion of correctly predicted observations relative to the total number of observations (Eq. 1). To avoid overfitting, the accuracy of the models was determined separately for the training and testing data. The lower the overfitting, the closer the accuracy of these two sets.
2. F1-score: It is the harmonic mean of precision and recall (Eq. 2). It is a more reliable metric than either precision or recall alone, especially for imbalanced datasets, where the number of positive and negative examples is not equal.

1 $$\:Accuracy=\:\frac{TN+TP}{TN+FP+TP+FN}$$

2 $$\:F1-score=2\left(\frac{Precision\times\:Recall}{Precision+\:Recall}\right);\:where,$$

$$\:Percision\:=\:\frac{TP}{TP+FP}\:,\:and\:Recall\:=\frac{TP}{TP+FN}$$

where $\:TP$ represents True Positive, denoting the count of positive examples that are correctly predicted as positive; $\:TN$ represents True Negative, is the count of negative examples that are correctly predicted as negative; $\:FP$ signifies False Positive, indicating the count of negative examples that are incorrectly predicted as positive, and finally, $\:FN$ denotes False Negatives, signifying the count of positive examples that are incorrectly predicted as negative.

c. Determining the effect of environmental and surveillance factors on the security perception of female cyclists. The SelectKBest algorithm^79^ from the Python Scikit-learn toolkit was used to determine the effect of environmental and surveillance factors on the security perception of female cyclists in the machine-learning models. SelectKBest algorithm identified the environmental and surveillance factors that were most important for predicting the security perception of female cyclists by selecting the K best features based on a given scoring function.

## Results and discussion

**Fig. 7:**
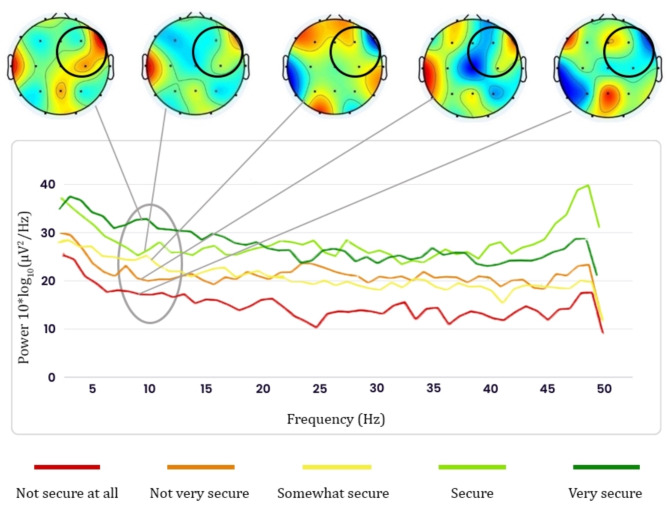
EEG power analysis on women’s security perception in the alpha frequency band for mean selected channels.

The findings of the clustering analysis of brain signals with sequential labeling in five levels of security perception of female cyclists (step 3.2) are presented in Fig. 7. Upon close examination of the color spectrum maps of the brain for each cluster, it became evident that when the level of security perception progresses from the “not secure at all” cluster to the “very secure” cluster, there is a noticeable shift towards red in the color spectrum surrounding the F4, F8, and C4 channels. This observation suggests a rise in the mean spectral power of the brainwave signal within the alpha frequency band (8–12 Hz) across these three channels. This particular feature was identified as the primary item throughout the feature selection process outlined in Step 3.1. Furthermore, the spectral power graphs associated with each cluster are also included in Fig. 7. These graphs illustrate the notable correlation between security perception levels and EEG signal clusters’ sequential arrangement. It can be observed that there is a positive correlation between the mean spectral power of the brainwave signal within the alpha frequency band and the level of security perception. In other words, the higher the level of security perception, the higher the value of the mean spectral power of the brainwave signal within the alpha frequency band. Therefore, these results suggest that we can use the sequential clustering of brain signals as labels expressing the perception of security of female cyclists when classifying environmental and surveillance factors.

Table 3 displays the evaluation metrics (according to Step 3.3.b) of the classification methods applied to the mean spectral power of the brainwave signal within the alpha frequency band regarding the four machine learning methods (RF, SVM, LR, and MLP) used in this study (outlined in step 3.3). A higher value of the F1-score indicates a greater level of reliability of the model^64^. Additionally, it is important to minimize the disparity between the accuracy of the train and test data, as substantial differences in the train and test accuracy could raise doubts about the model’s reliability and indicate a potential issue with overfitting^79^. It is evident from Table 3 that the SVM model, which exhibits an F1-score of 0.74, emerged as the most effective approach for classifying the environmental and surveillance factors. These findings indicate that the presented approach can be employed to classify environmental and surveillance factors that influence security perception.

**Table 3:** Results of classification models for brainwave power representing perceived security.

Method	F1_score	Train accuracy	Test accuracy
LR	0.69	0.680	0.690
RF	0.71	0.746	0.714
SVM	0.74	0.730	0.674
MLP	0.62	0.638	0.619

**Fig. 8:**
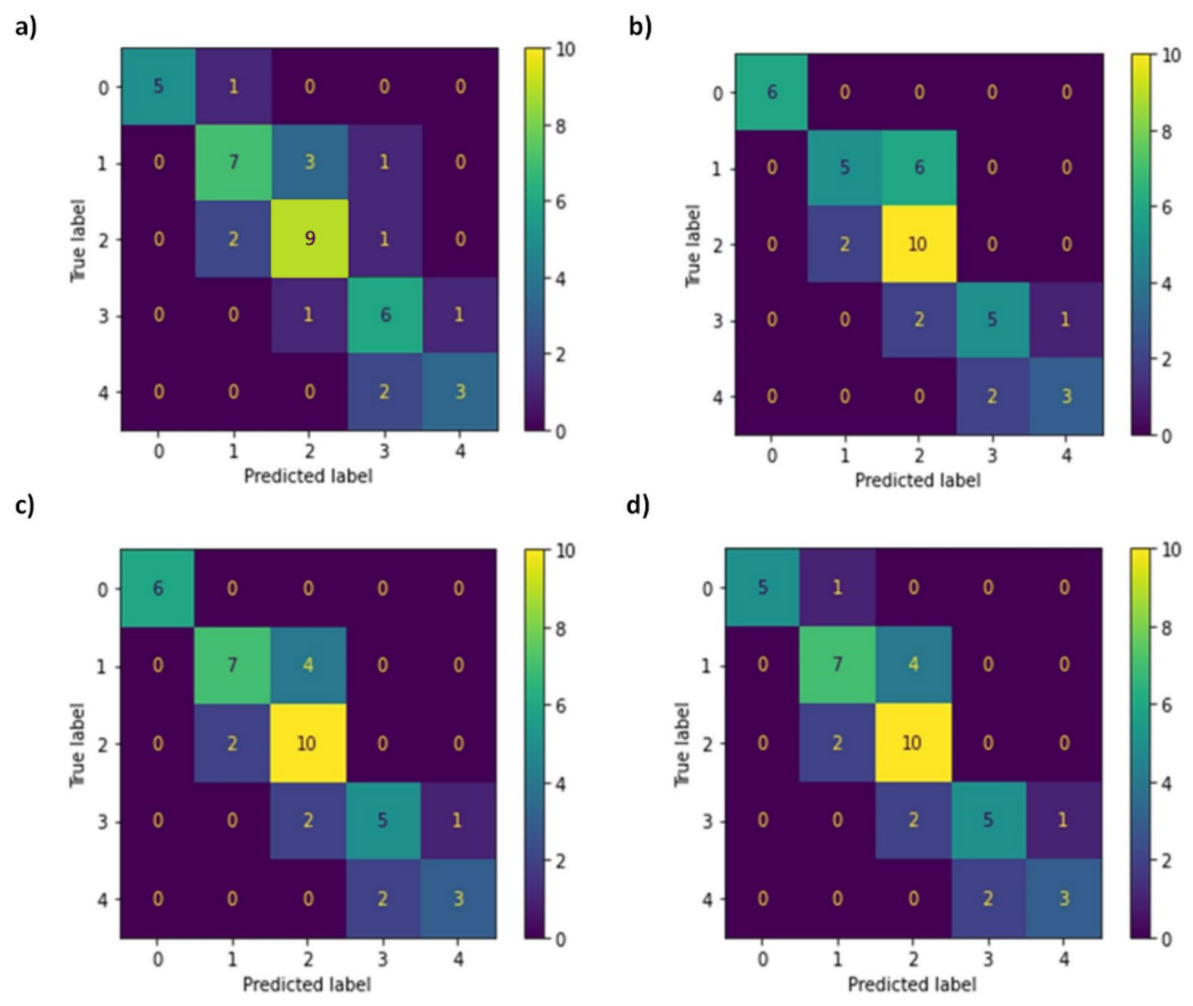
Confusion matrix for classification models for brainwave power representing perceived security; (**a**) RF, (**b**) LR, (**c**) SVM, and (**d**) MLP.

In addition to the aforementioned evaluation measures, the Confusion Matrix^80^ was employed as a visual tool for assessing the accuracy of classifications. Figure 8 displays the Confusion Matrix for the machine learning techniques employed in the classification of environmental and surveillance factors. The classification technique that exhibits bigger values along the matrix’s main diagonal is deemed more precise^68^. Like the outcomes of the F1-scores, the SVM model showed higher accuracy in classification models for brainwave power representing perceived security.

**Fig. 9:**
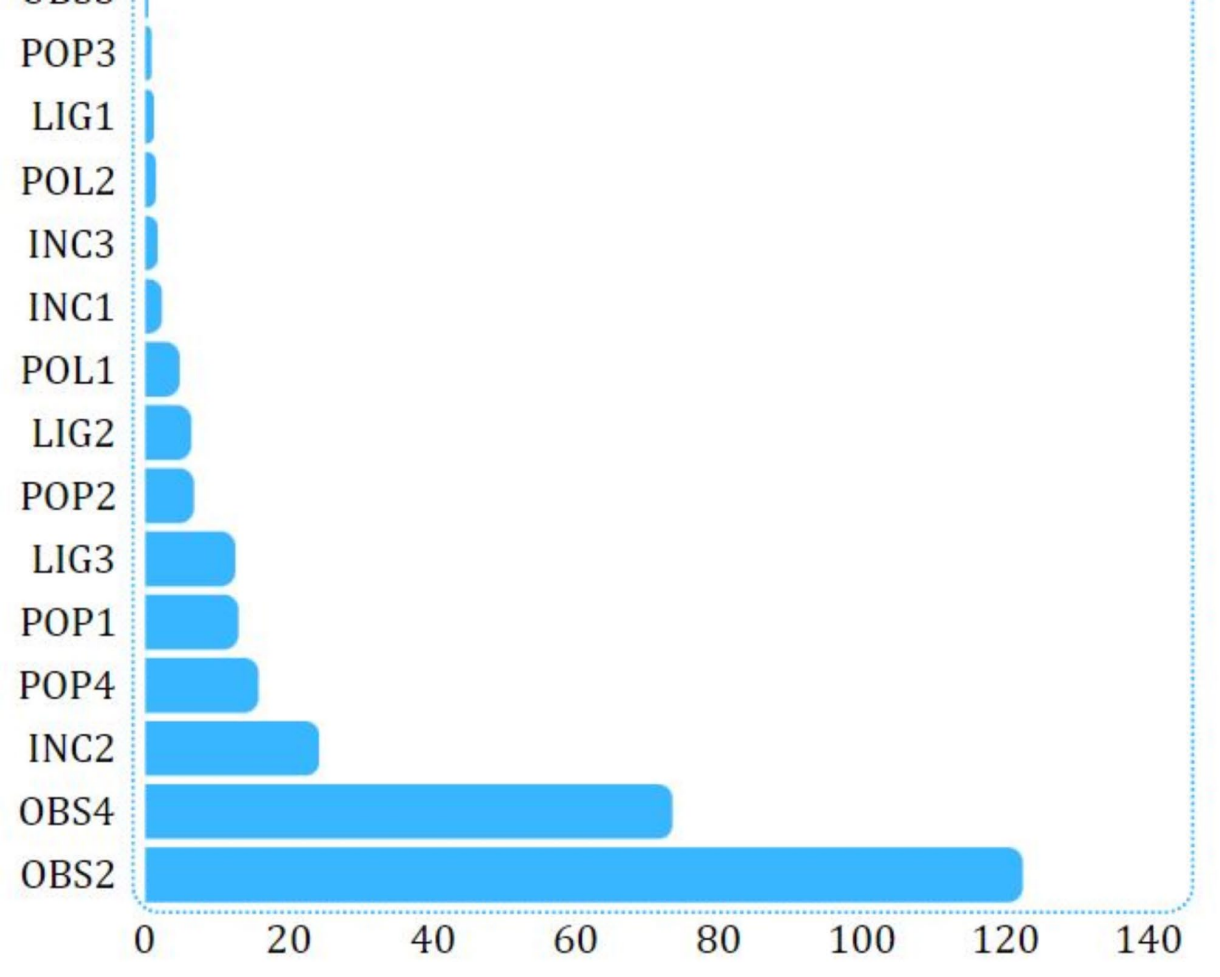
Relative influence of environmental and surveillance factors on female cyclist’s perceived security.

Finally, the SelectKBest algorithm was utilized to estimate the relative effect of environmental and surveillance factors on women cyclists’ perception of security based on the classification performed on the brainwave signals of the participants. The result of this algorithm has been shown in Fig. 9. In this figure, the presence of obstacles such as kiosks, passing the bicycle path through underpasses and tunnels, and passing the bicycle path through relatively decent areas of the city had the greatest effect in differentiating the classes of women cyclists’ perception of security. To put it better, these are the factors that produce the highest feeling of insecurity for female cyclists. Examining these results can provide a potential for managers, decision-makers, and planners to have a better awareness of the security status of cyclists, predominantly female cyclists. According to the findings of this research, in addition to improving the existing circumstances, better and more secure cycling routes can be constructed to encourage more citizens to ride bicycles as a clean and active transportation mode.

### Comparison with prior works

We have compared our study to other relevant research in the field of EEG signal classification within transportation contexts. Our study identified SVM as the best-performing algorithm with an accuracy of 67.4%. This finding aligns with several other studies in the field, where SVM has emerged as a top-performing algorithm. Sharma, et al.^81^ reported SVM as their best-performing algorithm with an accuracy of 87.0% for stress detection using EEG signals. Similarly, Kim, et al.^82^ found SVM to be the most effective for predicting driver’s EEG-based workload levels, achieving an accuracy of 84.3%. Halim and Rehan^83^ also identified SVM as their top performer, with an accuracy of 87.7% for identifying driving-induced stress patterns.

However, it is noteworthy to say that different studies have identified varying best-performing algorithms based on their specific contexts and datasets. Zhou, et al.^15^, for instance, found LR to be the most effective, with an accuracy of 92.9% for driving fatigue detection. In addition, Wang, et al.^26^ identified Bagging as their best performer, with an accuracy of 89.5% for evaluating driver fatigue.

The range of accuracies observed across these studies (from 67.4% in our study to 92.9% in Zhou, et al.^15^ study) underscores the influence of context-specific factors and dataset characteristics on algorithm performance. Our study’s focus on women’s perception of security while cycling in urban environments presents a unique context compared to the driving-related scenarios in other studies.

The consistency in SVM’s performance across multiple studies, including ours, suggests its robustness in handling various EEG signal classification tasks. However, the variability in best-performing algorithms across studies highlights the importance of testing multiple approaches for each specific application.

Our results, while showing lower accuracy compared to some driving-related studies, align with the general trends observed in the literature. The use of SVM and the testing of multiple algorithms (RF, SVM, LR, MLP) are consistent with methodologies employed in comparable research, reinforcing the validity of our approach within the unique context of our study. A summary of this comparison is presented in Table 4.

**Table 4:** Comparing the results of EEG-based classifiers in transportation-related studies.

Study	Context	Dataset	Algorithms used	Best performing algorithm
Current study	Examining factors influencing women’s perception of security while cycling in urban environments	A 208 × 105-dimensional dataset containing participants’ brainwave signals	RF, SVM, LR, MLP	SVM (accuracy = 67.4%)
Zhou, et al. ^15^	Driving fatigue detection by classifying EEG signals	EEG signal data from multiple subjects	SVM, DT, LR, RF, KNN, ADB, GBM	LR (accuracy = 92.9%)
Sharma, et al. ^81^	Stress detection by classifying EEG signals	Short-duration EEG signals	J48 decision trees, NB, RF, KNN, SVM	SVM (accuracy = 87.0%)
Halim and Rehan ^83^	Machine learning-based approach to identify driving-induced stress patterns by classifying EEG signals	EEG signals from 50 automotive drivers	SVM, NN, RF	SVM (accuracy = 87.7%)
Wang, et al. ^26^	Developing a high-performance, stable system for evaluating driver fatigue by classifying EEG signals	EEG signals from 12 healthy adult subjects	Bagging, Boosting, KNN, SVM, DT, RF	Bagging (accuracy = 89.5%)
Kim, et al. ^82^	Predicting driver’s EEG-based workload levels using vehicle driving information	Field-of-test data collected from driving on actual roads	SVM	SVM (accuracy = 84.3%)

### Limitation

This study has several limitations that warrant consideration. Our research focused exclusively on female cyclists in Tehran, with a limited sample size due to laboratory testing constraints. This restriction may have impacted on the precision of our findings and precluded the use of more advanced analytical techniques, such as deep learning algorithms. Additionally, the specificity of our sample may limit the generalizability of our findings to other demographics or geographical locations.

While efforts were made to create an immersive VR environment simulating Tehran settings, inherent differences between virtual and physical environments persist. Participants’ awareness of being in a simulated environment may have induced bias, potentially affecting their reactions and sense of security compared to real-world conditions. Technological limitations of both VR and EEG equipment, including graphics quality, hardware constraints, signal noise, and EEG sensor sensitivity, may have influenced the level of immersion, realism, and data quality. These factors collectively contribute to a potential discrepancy between the simulated experience and real-world cycling conditions in Tehran.

Our study did not account for several factors that could influence security perception, including weather conditions, individual characteristics, personal past experiences, and cultural factors. The complex interplay of these variables in real-world settings may yield different results from those observed in our controlled environment. Future research should address these limitations by expanding the sample size and demographic diversity, enhancing VR technology to increase realism and immersion, incorporating additional variables to provide a more comprehensive understanding of factors influencing security perception among cyclists, and conducting comparative studies across different urban environments and cultural contexts.

### Practical implication: spatiotemporal performance measure

Using Tehran as a case study, this study developed a machine learning model capable of effectively classifying the environmental and surveillance factors that influence female cyclists’ perception of security. This model enables the development of an online dashboard that visualizes a spatiotemporal dynamic heatmap representing the perceived security level of women inside a city’s bicycle network. Through the examination of the factors affecting the perception of security across locations in a network and the computation of the perceived security classification, decision-makers can gain valuable insights into the temporal and spatial variations of perceived security. The advancement of this technology will allow for better management of the bicycle network. A preliminary demonstration of this task has been conducted, and its details will be expounded upon in the following part.

**Fig. 10:**
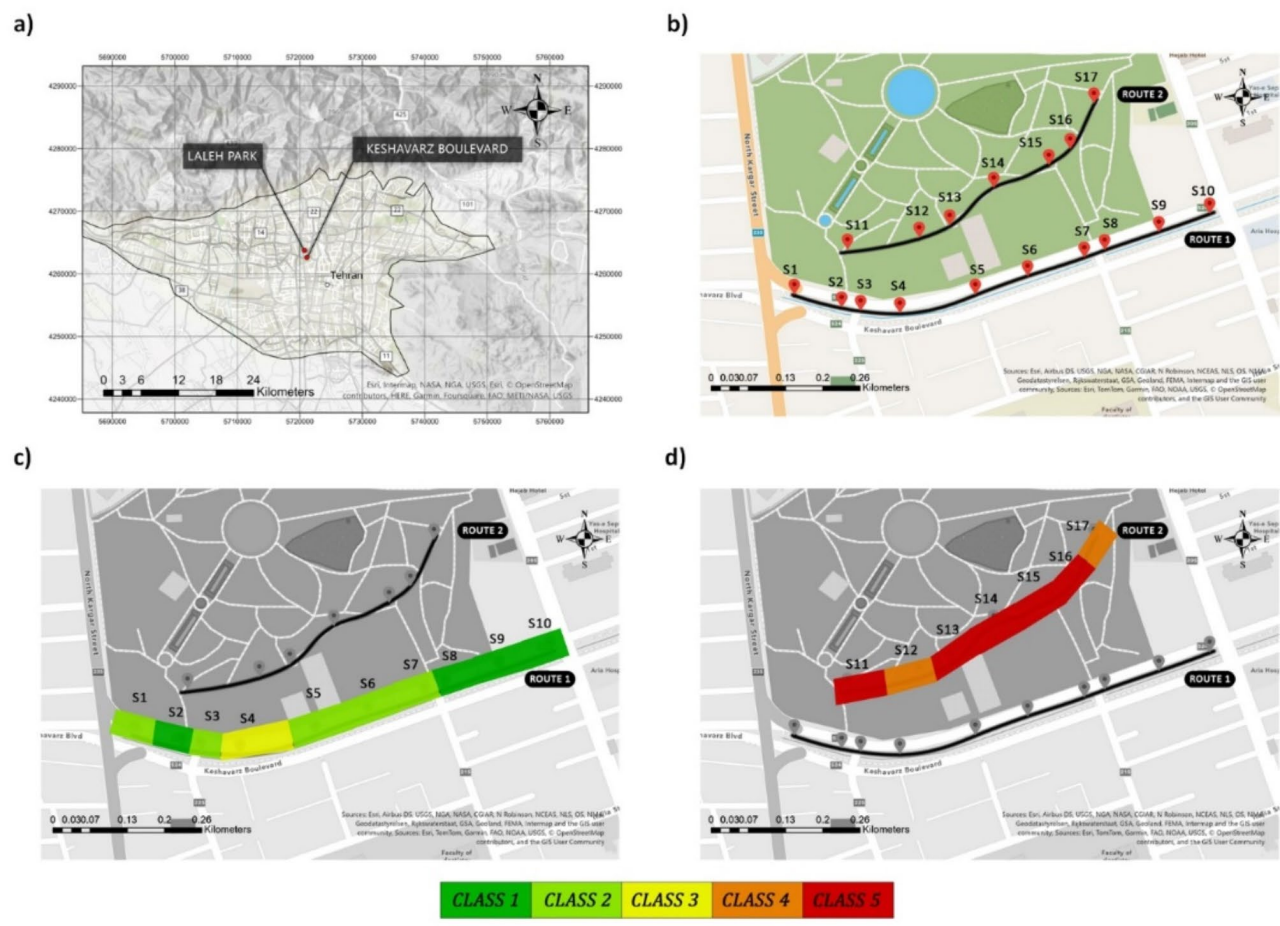
Spatiotemporal analysis in Laleh Park and Keshavarz Boulevard in Tehran on August 30, 2022. Maps generated in the ArcGIS Pro 2.8.6 software (www.esri.com): (**a**) Tehran city and the location of the two cycling routes considered in this study; (**b**) location of the stations; (**c**) classification heatmap of women’s perceived security of Route 1 at Keshavarz boulevard between 19:15 and 19:30; and (**d**) classification heatmap of women’s perceived security of Route 2 at Laleh Park between 21:45 and 22:00.

Initially, two cycling routes were chosen within the central business district of Tehran, as seen in Fig. 10a, b. Subsequently, a limited number of stations were carefully chosen inside each route. The precise location data of the selected stations was collected using advanced “Raymax Ultimate GNSS Receiver” mapping equipment. In addition, the data for the environmental and surveillance parameters were collected at 15-minute intervals. Then, the data gathered from the stations was input into ArcGIS Pro 2.8.6^84^ software, which was used to create the maps shown in Fig. 10. Subsequently, using the machine learning model developed in the current study, the classification of factors influencing security perception was done using real data from the stations. The heatmaps illustrating the perceived security levels of women along the two designated routes are presented in Fig. 10c, d. Figure 10c displays the classification heatmap for route 1, encompassing stations S1 to S10, while Fig. 10d exhibits the classification heatmap for route 2, encompassing stations S11 to S17. The heatmaps presented in this analysis designate the classes denoted from “not secure at all” to “very secure” as “Class 1” to “Class 5,” respectively. It should be mentioned that Route 1 is a busy road, and data was collected during a period of high traffic flow and in daylight settings. However, path 2 can be characterized as a bicycle path with low traffic activity located within a park. It is worth noting that the data collection for this particular route occurred during nighttime hours (for details on the settings of each station, see Supplementary Table S2).

Figure 10 also depicts the effect of the presence of informal surveillance and the time of day on women’s perceptions of security. As you can see, the heat map of Route 1, which was captured in daylight and runs through a busy route, is mostly green. The heat map of Route 2, which travels through a low-traffic park and data collecting was done at night, is mostly red. Looking closely at stations S11 and S12, it is clear that the presence of police officers, as an indication of formal surveillance, raises the level of security perception from class 1 to class 2. Furthermore, a comparison of stations S2 and S4 demonstrates that the existence of physical obstacles, such as kiosks and the absence of police officers, has a substantial effect on the level of security perception, resulting in a drop from class 5 to class 3. The results of this study show that implementing measures aimed at reducing obstacles, increasing formal surveillance, and improving the lighting conditions of bicycle routes could enhance female cyclists’ perception of security.

What has been described in this section is an example of the practical implications of the research findings. Only a small part of Tehran’s cycling network was explored offline in this study, but this model has the potential to become an online real-time dashboard for regulating and monitoring a city’s whole cycling network. Future development of this tool may aid in improving cycling infrastructure to meet women’s preferences, hence increasing their participation in using sustainable and active modes of transportation. The authors of this article are actively conducting research on this subject.

## Conclusion

This study aimed to investigate the security perception of female cyclists in Tehran by utilizing EEG data, a VR simulator, and machine learning techniques. The findings demonstrate a correlation between the spectral power of brainwave signals in the alpha frequency band and the perceived security levels of the participants. Specifically, as the perceived security level increased, there was a corresponding rise in the mean spectral power within the alpha frequency band. This correlation was identified through the clustering analysis of brain signals and validated by the performance of various classification models, with the SVM model showing the highest accuracy.

The analysis revealed that environmental and surveillance factors impact the security perception of female cyclists. The presence of formal surveillance, such as police officers, and the reduction of physical obstacles, like kiosks, were found to enhance the perception of security. Conversely, poorly lit areas and the absence of surveillance were associated with lower security perceptions. These insights suggest that improving the cycling infrastructure, including better lighting and increased formal surveillance, can positively influence female cyclists’ sense of security.

The practical implications of this research are substantial. The model developed in this study has the potential to be expanded into a dashboard for monitoring and regulating the entire cycling network of a city. Such a tool could assist urban planners and policymakers in designing cycling infrastructures that cater to women’s preferences, thereby promoting their participation in cycling as a sustainable and active mode of transportation. The authors are actively pursuing further research to refine and enhance this model for broader applications.

In conclusion, this study provides valuable insights into the factors influencing the security perception of female cyclists. The integration of EEG data with machine learning techniques offers a novel approach to understanding and improving urban cycling environments. Future research should focus on increasing the sample size and incorporating diverse demographic groups to enhance the generalizability of the findings. Moreover, continuous development of real-time monitoring tools will be essential in creating safer and more inclusive cycling networks.

## Electronic supplementary material

Below is the link to the electronic supplementary material.

Supplementary Material 1

## Data Availability

The dataset is freely available at: To access it, visit 10.6084/m9.figshare.25211705. This dataset includes EEG recordings, VR simulation logs, details of the machine learning (ML) techniques, and survey responses collected from 52 female cyclists in Tehran, Iran, to study factors influencing their perception of security while cycling. EEG data was gathered using a 21-channel wireless EEG headset during VR bicycle simulations depicting various environmental settings. The Python code for clustering, used to apply ML techniques in this study, is also provided. The VR logs capture details about the simulated cycling routes and conditions, while the survey responses provide self-reported assessments of perceived security corresponding to each simulation.The data is being made available under the terms of the Apache License 2.0, which allows anyone to freely use, distribute, and modify the data for any purpose as long as the terms of the license are retained. Proper attribution must be given to the original authors. The data may be used to reproduce the analysis described in the study summary or for any other academic, commercial, or personal purposes without restriction. We hope that releasing this data will allow others to study further factors impacting women’s cycling security.

## References

[1] da Silva, C., da Silva, D. R. & Nélson, A. Sustainable modes and violence: Perceived safety and exposure to crimes on trips to and from a Brazilian university campus. *J. Transp. Health*. **16**, 100817 (2020).

[2] Firoozi Yeganeh, S., Khademi, N., Farahani, H. & Besharat, M. A. A qualitative exploration of factors influencing women’s intention to use shared taxis: a study on the characteristics of urban commuting behavior in Iran. *Transp. Policy*. **129**, 90–104 (2022).

[3] Hsu, H. P. How does fear of sexual harassment on transit affect women’s use of transit? In *Women’s Issues in Transportation, Summary of the 4th International Conference*, vol. 2, 85–94 (2009).

[4] Dunckel-Graglia, A. & Women-Only, T. How pink public transportation changes public perception of women’s mobility. *J. Public. Transp.* **16**, 85–105 (2013).

[5] Gardner, N., Cui, J. & Coiacetto, E. Harassment on public transport and its impacts on women’s travel behaviour. *Aust. Plan.* **54**, 8–15 (2017).

[6] Stark, J. & Meschik, M. Women’s everyday mobility: frightening situations and their impacts on travel behaviour. *Transp. Res. Part. F: Traffic Psychol. Behav.* **54**, 311–323 (2018).

[7] Kash, G. Always on the defensive: the effects of Transit sexual assault on travel behavior and experience in Colombia and Bolivia. *J. Transp. Health*. **13**, 234–246 (2019).

[8] Orozco-Fontalvo, M., Soto, J., Arévalo, A. & Oviedo-Trespalacios, O. Women’s perceived risk of sexual harassment in a Bus Rapid Transit (BRT) system: the case of Barranquilla, Colombia. *J. Transp. Health*. **14**, 100598 (2019).

[9] Quinones, L. M. Sexual harassment in public transport in Bogotá. *Transp. Res. Part. A Policy Pract.* **139**, 54–69 (2020).

[10] DDCT. Statistical yearbook of active travel modes in Tehran. Transport and Traffic Organization, Transport and Traffic Deputy of Tehran Municipality, Tehran, Iran 1 (2022) (**in Persian**).

[11] Di Flumeri, G. et al. EEG-based mental workload neurometric to evaluate the impact of different traffic and road conditions in real driving settings. *Front. Hum. Neurosci.* **12** (2018). 10.3389/fnhum.2018.00509 PMC6305466 30618686

[12] Cao, Z., Chuang, C. H., King, J. K. & Lin, C. T. Multi-channel EEG recordings during a sustained-attention driving task. *Sci. Data*. **6**, 19 (2019). 30952963 10.1038/s41597-019-0027-4 PMC6472414

[13] Arefnezhad, S. et al. Driver drowsiness estimation using EEG signals with a dynamical encoder–decoder modeling framework. *Sci. Rep.* **12**, 2650 (2022). 35173189 10.1038/s41598-022-05810-x PMC8850607

[14] Doudou, M., Bouabdallah, A. & Berge-Cherfaoui, V. Driver drowsiness measurement technologies: current research, market solutions, and challenges. *Int. J. Intell. Transp. Syst. Res.* **18**, 297–319 (2020).

[15] Zhou, Y., Zeng, C. & Mu, Z. Optimal feature-algorithm combination research for EEG fatigue driving detection based on functional brain network. *IET Biom.* **12**, 65–76 (2023).

[16] Begum, S., Barua, S. & Ahmed, M. In-Vehicle stress monitoring based on EEG Signal. *Int. J. Eng. Res. Appl.* **07**, 55–71 (2017).

[17] Shin, J. H. et al. Wearable EEG electronics for a brain–AI closed-loop system to enhance autonomous machine decision-making. *NPJ Flex. Electron.* **6**, 32 (2022).

[18] Akhtar, M. & Moridpour, S. A. Review of traffic congestion prediction using artificial intelligence. *J. Adv. Transp.* **2021**, 8878011 (2021).

[19] Omrani, H. Predicting travel mode of individuals by machine learning. *Transp. Res. Proc.*. **10**, 840–849 (2015).

[20] Tang, T., Liu, R., Choudhury, C., Fonzone, A. & Wang, Y. Predicting hourly boarding demand of bus passengers using imbalanced records from smart-cards: a deep learning approach. *IEEE Trans. Intell. Transp. Syst.* **24**, 5105–5119 (2023).

[21] Yang, S. et al. What contributes to driving behavior prediction at unsignalized intersections? *Transp. Res. Part. C Emerg. Technol.* **108**, 100–114 (2019).

[22] Le, H. T. K., West, A., Quinn, F. & Hankey, S. Advancing cycling among women: an exploratory study of north American cyclists. *J. Transp. Land. Use* **12** (2019).

[23] Kim, E. J. Analysis of travel mode choice in seoul using an interpretable machine learning approach. *J. Adv. Transp.* 1–13 (2021). (2021).

[24] Balam, V. P., Sameer, V. U. & Chinara, S. Automated classification system for drowsiness detection using convolutional neural network and electroencephalogram. *IET Intel. Transport Syst.* **15**, 514–524 (2021).

[25] Hu, S., Zheng, G. & Peters, B. Driver fatigue detection from electroencephalogram spectrum after electrooculography artefact removal. *IET Intel. Transport Syst.* **7**, 105–113 (2012).

[26] Wang, P., Min, J. & Hu, J. Ensemble classifier for driver’s fatigue detection based on a single EEG channel. *IET Intel. Transport Syst.* **12**, 1322–1328 (2018).

[27] Zink, R., Hunyadi, B., Huffel, S. & de Vos, M. Mobile EEG on the bike: disentangling attentional and physical contributions to auditory attention tasks. *J. Neural Eng.* **13**, 046017 (2016). 27351459 10.1088/1741-2560/13/4/046017

[28] Chiodi, S. Crime prevention through urban design and planning in the smart city era: the challenge of disseminating CP-UDP in Italy: learning from Europe. *J. Place Manag. Dev.* **9**, 137–152 (2016).

[29] Navarrete-Hernandez, P., Vetro, A. & Concha, P. Building safer public spaces: exploring gender difference in the perception of safety in public space through urban design interventions. *Landsc. Urban Plann.* **214**, 104180 (2021).

[30] Eurocultures, Architektinnen, F. O. & Vie, P. v. G. C. d., Praxis & Seirov-Nirov. *European Charter for Women in the City: Moving Towards a Gender-conscious City: a Common Platform for Discussion at European Level : Parity in Democracy Will Improve Living Conditions for All* (European Commission, Equal Opportunities Unit, 1994).

[31] Mahrous, A., Moustafa, Y. & Abou El-Ela, M. Physical characteristics and perceived security in urban parks: investigation in the Egyptian context. *Ain Shams Eng. J.* **9** (2018).

[32] Anciaes, P. R. & Jones, P. Estimating preferences for different types of pedestrian crossing facilities. *Transp. Res. Part. F Traffic Psychol. Behav.* **52**, 222–237 (2018).

[33] Khademi, N. et al. Building a less intimidating cycling environment for women: a structural equation modeling analysis based on a VR-based laboratory experiment. *Transp. Res. Part. F Traffic Psychol. Behav.* **100**, 431–457 (2024).

[34] Adu-Mireku, S. Fear of crime among residents of three communities in Accra, Ghana. *Int. J. Comp. Sociol. ***43**, 153–168 (2002).

[35] Paydar, M., Kamani-Fard, A. & Etminani, R. Perceived security of women in relation to their path choice toward sustainable neighborhood in Santiago, Chile. *Cities* **60** (2017).

[36] Wilson, J. Q. & Kelling, G. L. Broken windows. *Atl. Monthly*. **249**, 29–38 (1982).

[37] Foster, S., Giles-Corti, B. & Knuiman, M. Does fear of crime discourage walkers? A social-ecological exploration of fear as a deterrent to walking. *Environ. Behav.* **46**, 698–717 (2014).

[38] Gargiulo, I. et al. Women’s safety perception assessment in an urban stream corridor: developing a safety map based on qualitative GIS. *Landsc. Urban Plann.* **198**, 103779 (2020).

[39] Jacobs, J. *The Death and Life of Great American Cities* (Random House, 1961).

[40] Newman, O. *Defensible Space; Crime Prevention through Urban Design* (Macmillan, 1972).

[41] Hillier, B. *Space is the Machine: A Configurational Theory of Architecture* (Cambridge University Press, 1996).

[42] Ding, H., Loukaitou-Sideris, A. & Agrawal, A. Sexual harassment and assault in Transit environments: a review of the English-language literature. *J. Plann. Lit.*. **35**, 088541222091112 (2020).

[43] Basu, N., Haque, S. M. M., King, M., Kamruzzaman, M. & Oviedo-Trespalacios, O. The unequal gender effects of the suburban built environment on perceptions of security. *J. Transp. Health*. **23**, 101243 (2021).

[44] Ceccato, V., Gaudelet, N. & Graf, G. Crime and safety in transit environments: a systematic review of the English and the French literature, 1970–2020. *Public. Transp.* **14** (2022).

[45] Börjesson, M. Valuing perceived insecurity associated with use of and access to public transport. *Transp. Policy*. **22**, 1–10 (2012).

[46] Gekoski, A. & Gray Jacqueline, Horvath, Miranda, Edwards, Sarah, Emirali, Aliye, Adler, Joanna. *‘What Works’ in Reducing Sexual Harassment and Sexual Offences on Public Transport Nationally and Internationally: A Rapid Evidence Assessment* (2015).

[47] Loukaitou-Sideris, A. In *Safety and Security in Transit Environments: An Interdisciplinary Approach* (eds Vania, C. & Andrew, N.) 291–308 (Palgrave Macmillan, 2015).

[48] Noorbakhsh, P., Khademi, N. & Chaiyasarn, K. Exploration of women cyclists’ perceived security using tree-based machine learning algorithms. *Proc. Comput. Sci.* **220**, 624–631 (2023).

[49] Fernandez Abenoza, R., Ceccato, V., Susilo, Y., Cats, O. Individual travel, and bus stop characteristics influencing travelers’ safety perceptions. *Transp. Res. Record J. Transp. Res. Board.* **2672** (2018).

[50] Su, D., Nguyen-Phuoc, D. & Johnson, L. Effects of perceived safety, involvement and perceived service quality on loyalty intention among ride-sourcing passengers. *Transportation* **48** (2021).

[51] Witmer, B. G. & Singer, M. J. Measuring presence in virtual environments: a presence questionnaire. *Presence: Teleoperators Virtual Environ.* **7**, 225–240 (1998).

[52] Bogacz, M. et al. Comparison of cycling behavior between keyboard-controlled and instrumented bicycle experiments in virtual reality. *Transp. Res. Rec.* **2674**, 244–257 (2020).

[53] Shie, Q. & Dapang, C. Joint time-frequency analysis. *IEEE. Signal. Process. Mag.* **16**, 52–67 (1999).

[54] Christiano, L. J. & Fitzgerald, T. J. The band pass filter. *Int. Econ. Rev.* **44**, 435–465 (2003).

[55] Repovš, G. Dealing with noise in EEG recording and data analysis. *Inform. Med. Slov.* **15**, 18–25 (2010).

[56] Jiang, X., Bian, G. B. & Tian, Z. Removal of artifacts from EEG signals: a review. *Sensors* **19**, 987 (2019). 30813520 10.3390/s19050987 PMC6427454

[57] Berg, P. & Scherg, M. A multiple source approach to the correction of eye artifacts. *Electroencephalogr. Clin. Neurophysiol.* **90**, 229–241 (1994). 7511504 10.1016/0013-4694(94)90094-9

[58] Ille, N., Berg, P. & Scherg, M. Artifact correction of the Ongoing EEG using spatial filters based on artifact and brain signal topographies. *J. Clin. Neurophysiol.* **19**, 113–124 (2002). 11997722 10.1097/00004691-200203000-00002

[59] Delorme, A. & Makeig, S. EEGLAB: an open source toolbox for analysis of single-trial EEG dynamics including independent component analysis. *J. Neurosci. Methods*. **134**, 9–21 (2004). 15102499 10.1016/j.jneumeth.2003.10.009

[60] Palva, S. & Palva, J. M. New vistas for α-frequency band oscillations. *Trends Neurosci.* **30**, 150–158 (2007). 17307258 10.1016/j.tins.2007.02.001

[61] Yeom, S., Kim, H. & Hong, T. Psychological and physiological effects of a green wall on occupants: a cross-over study in virtual reality. *Build. Environ.* **204**, 108134 (2021).

[62] Roth, S. & Cohen, L. J. Approach, avoidance, and coping with stress. *Am. Psychol.* **41**, 813 (1986). 3740641 10.1037//0003-066x.41.7.813

[63] Choi, Y., Kim, M. & Chun, C. Measurement of occupants’ stress based on electroencephalograms (EEG) in twelve combined environments. *Build. Environ.* **88**, 65–72 (2015).

[64] Chatterjee, D., Gavas, R. & Saha, S. K. Detection of mental stress using novel spatio-temporal distribution of brain activations. *Biomed. Signal Process. Control*. **82**, 104526 (2023).

[65] Teplan, M. Fundamental of EEG measurement. *Meas. Sci. Rev.* **2** (2002).

[66] Chaddad, A., Wu, Y., Kateb, R. & Bouridane, A. Electroencephalography signal processing: a comprehensive review and analysis of methods and techniques. *Sensors* **23** (2023). 10.3390/s23146434 PMC10385593 37514728

[67] Welch, P. The use of fast Fourier transform for the estimation of power spectra: a method based on time averaging over short, modified periodograms. *IEEE Trans. Audio Electroacoust.* **15**, 70–73 (1967).

[68] Quackenbush, J. Microarray data normalization and transformation. *Nat. Genet.* **32**, 496–501 (2003). 10.1038/ng1032 12454644

[69] Singh, D. & Singh, B. Investigating the impact of data normalization on classification performance. *Appl. Soft Comput.* 105524 (2019).

[70] Dolcos, F. et al. Neural correlates of emotion-attention interactions: from perception, learning, and memory to social cognition, individual differences, and training interventions. *Neurosci. Biobehav. Rev.*. **108**, 559–601 (2020). 31446010 10.1016/j.neubiorev.2019.08.017

[71] Lin, C. T. et al. Real-time EEG signal enhancement using canonical correlation analysis and Gaussian mixture clustering. *J. Healthc. Eng.* **2018**, 5081258 (2018). 10.1155/2018/5081258 PMC5823426 29599950

[72] Mendonça, F., Mostafa, S. S. & Morgado-Dias, F., Ravelo-García, A. G. Cyclic alternating pattern estimation based on a probabilistic model over an EEG signal. *Biomed. Signal Process. Control*. **62**, 102063 (2020).

[73] Fu, R., Li, Z. & Wang, J. An optimized GMM algorithm and its application in single-trial motor imagination recognition. *Biomed. Signal Process. Control*. **72**, 103327 (2022).

[74] Arı, Ç., Aksoy, S. & Arıkan, O. Maximum likelihood estimation of Gaussian mixture models using stochastic search. *Pattern Recogn.* **45**, 2804–2816 (2012).

[75] Coraggio, L. & Coretto, P. Selecting the number of clusters, clustering models, and algorithms. A unifying approach based on the quadratic discriminant score. *J. Multivar. Anal.* **196**, 105181 (2023).

[76] Bergstra, J. & Bengio, Y. Random search for hyper-parameter optimization. *J. Mach. Learn. Res.* **13**, 281–305 (2012).

[77] Refaeilzadeh, P., Tang, L. & Liu, H. In *Encyclopedia of Database Systems* (eds Ling, L. & Tamer ÖZsu, M.) 532–538 (Springer US, 2009).

[78] Shalev-Shwartz, S. & Ben-David, S. *Understanding Machine Learning: from Theory to Algorithms* (Cambridge University Press, 2014).

[79] Bonaccorso, G. *Machine Learning Algorithms* (Packt Publishing, 2017).

[80] Aggarwal, C. C. *Neural Networks and Deep Learning: A Textbook* (Springer International Publishing, 2018).

[81] Sharma, L. D. et al. Evolutionary inspired approach for mental stress detection using EEG signal. *Expert Syst. Appl.* **197**, 116634 (2022).

[82] Kim, H. S., Yoon, D., Shin, H. S. & Park, C. H. Predicting the EEG level of a driver based on driving information. *IEEE Trans. Intell. Transp. Syst.* **20**, 1215–1225 (2018).

[83] Halim, Z. & Rehan, M. On identification of driving-induced stress using electroencephalogram signals: a framework based on wearable safety-critical scheme and machine learning. *Inform. Fusion*. **53**, 66–79 (2020).

[84] ESRI. *ArcGIS Pro (Version 2.8.6) [Software]* (Environmental Systems Research Institute, 2021).

