# Supplementary material for: Brain activity patterns reflecting security perceptions of female cyclists in virtual reality experiments
Source: Sci Rep. 2025 Jan 4;15:761. doi: 10.1038/s41598-024-81271-8 (PMC11700102; doi:10.1038/s41598-024-81271-8)
Supplement: Supplementary file 1 — Supplementary Material 1 [file 41598_2024_81271_MOESM1_ESM.docx]

Supplementary information for: Brain activity patterns reflecting security perceptions of female cyclists in virtual reality experiments

Mohammad Arbabpour Bidgoli^a^, Arian Behmanesh^a^, Navid Khademi^a^, Phromphat Thansirichaisree^b*^, Zuduo Zheng^c^, Sara Saberi Moghadam Tehrani^d^, Sajjad Mazloum^a^, Sirisilp Kongsilp^e^

^a^ *School of Civil Engineering, College of Engineering, University of Tehran, Tehran, Iran.*

^b^ *Thammasat School of Engineering, Faculty of Engineering, Thammasat University Rangsit, Klong Luang, Pathumthani, Thailand.*

^c^ *School of Civil Engineering, Faculty of Engineering, Architecture and Information Technology, The University of Queensland, Australia.*

^d^ *Department of Physics, KTH Royal Institute of Technology, Stockholm, Sweden.*

^e^ *Department of Computer Engineering, Faculty of Engineering, Kasetsart University, Bangkok, Thailand.*

^*^ *Corresponding author’s e–mail: ckrisada@engr.tu.ac.th.*

Supplementary Tables

| Scenario | Time of day and street lighting | Obstacles, vegetation, and underpass | Evidence of incivility | Formal surveillance (presence of police) | Informal surveillance (presence of others) |
| --- | --- | --- | --- | --- | --- |
| **1** | LIG3 | OBS2 | INC2 | POL1 | POP2 |
| **2** | LIG2 | OBS1 | INC2 | POL2 | POP3 |
| **3** | LIG3 | OBS4 | INC3 | POL2 | POP1 |
| **4** | LIG1 | OBS3 | INC1 | POL1 | POP4 |
| **5** | LIG3 | OBS4 | INC1 | POL1 | POP3 |
| **6** | LIG1 | OBS1 | INC2 | POL2 | POP1 |
| **7** | LIG2 | OBS3 | INC1 | POL2 | POP2 |
| **8** | LIG1 | OBS2 | INC3 | POL1 | POP4 |
| **9** | LIG1 | OBS2 | INC1 | POL2 | POP3 |
| **10** | LIG2 | OBS4 | INC2 | POL1 | POP4 |
| **11** | LIG3 | OBS3 | INC2 | POL2 | POP1 |
| **12** | LIG1 | OBS1 | INC3 | POL1 | POP2 |
| **13** | LIG3 | OBS1 | INC1 | POL2 | POP4 |
| **14** | LIG2 | OBS2 | INC1 | POL1 | POP1 |
| **15** | LIG3 | OBS3 | INC3 | POL2 | POP3 |
| **16** | LIG1 | OBS4 | INC2 | POL1 | POP2 |

**Supplementary Table S1.** Experiment scenarios.

. . . . . . . . . . . . . . . . . . . . . . . . . . . . . . . . . . . . . . . . . . . . . . . . . . . . . . . . . . . . . . . . . . . . . . . . . . . . . . . . . . . . . . . . . . . . . . . . .

| Route | Point | Period | Time of the day  and street  lighting | Obstacles, vegetation, and underpass | Evidence of  incivility | Formal  surveillance | Informal  surveillance | **Perceived**  **security class** | Real view | Color index of security perception |
| --- | --- | --- | --- | --- | --- | --- | --- | --- | --- | --- |
| 1 | S1 | 19:15 – 19:30 | LIG1 | OBS1 | INC2 | POL2 | POP4 | CLASS 4 | Supplementary Figure S3.1 |  |
| 1 | S2 | 19:15 – 19:30 | LIG1 | OBS1 | INC2 | POL1 | POP4 | CLASS 5 | Supplementary Figure S3.2 |  |
| 1 | S3 | 19:15 – 19:30 | LIG1 | OBS1 | INC2 | POL2 | POP4 | CLASS 4 | Supplementary Figure S3.3 |  |
| 1 | S4 | 19:15 – 19:30 | LIG1 | OBS2 | INC2 | POL2 | POP4 | CLASS 3 | Supplementary Figure S3.4 |  |
| 1 | S5 | 19:15 – 19:30 | LIG1 | OBS1 | INC2 | POL2 | POP4 | CLASS 4 | Supplementary Figure S3.5 |  |
| 1 | S6 | 19:15 – 19:30 | LIG1 | OBS1 | INC2 | POL2 | POP4 | CLASS 4 | Supplementary Figure S3.6 |  |
| 1 | S7 | 19:15 – 19:30 | LIG1 | OBS1 | INC2 | POL2 | POP4 | CLASS 4 | Supplementary Figure S3.7 |  |
| 1 | S8 | 19:15 – 19:30 | LIG1 | OBS1 | INC2 | POL1 | POP4 | CLASS 5 | Supplementary Figure S3.8 |  |
| 1 | S9 | 19:15 – 19:30 | LIG1 | OBS1 | INC2 | POL2 | POP4 | CLASS 5 | Supplementary Figure S3.9 |  |
| 1 | S10 | 19:15 – 19:30 | LIG1 | OBS1 | INC2 | POL2 | POP4 | CLASS 5 | Supplementary Figure S3.10 |  |
| 2 | S11 | 21:45 – 22:00 | LIG2 | OBS1 | INC2 | POL2 | POP1 | CLASS 1 | Supplementary Figure S3.11 |  |
| 2 | S12 | 21:45 – 22:00 | LIG2 | OBS1 | INC2 | POL1 | POP1 | CLASS 2 | Supplementary Figure S3.12 |  |
| 2 | S13 | 21:45 – 22:00 | LIG2 | OBS2 | INC2 | POL2 | POP2 | CLASS 1 | Supplementary Figure S3.13 |  |
| 2 | S14 | 21:45 – 22:00 | LIG3 | OBS1 | INC2 | POL2 | POP1 | CLASS 1 | Supplementary Figure S3.14 |  |
| 2 | S15 | 21:45 – 22:00 | LIG3 | OBS1 | INC2 | POL2 | POP1 | CLASS 1 | Supplementary Figure S3.15 |  |
| 2 | S16 | 21:45 – 22:00 | LIG3 | OBS3 | INC2 | POL2 | POP2 | CLASS 1 | Supplementary Figure S3.16 |  |
| 2 | S17 | 21:45 – 22:00 | LIG2 | OBS1 | INC2 | POL2 | POP2 | CLASS 2 | Supplementary Figure S3.17 |  |

**Supplementary Table S2.** Classification of the perceived security in selected stations of the Laleh Park and Keshavarz Boulevard in Tehran on August 30, 2022.

. . . . . . . . . . . . . . . . . . . . . . . . . . . . . . . . . . . . . . . . . . . . . . . . . . . . . . . . . . . . . . . . . . . . . . . . . . . . . . . . . . . . . . . . . . . . . . . . .

Supplementary Figures


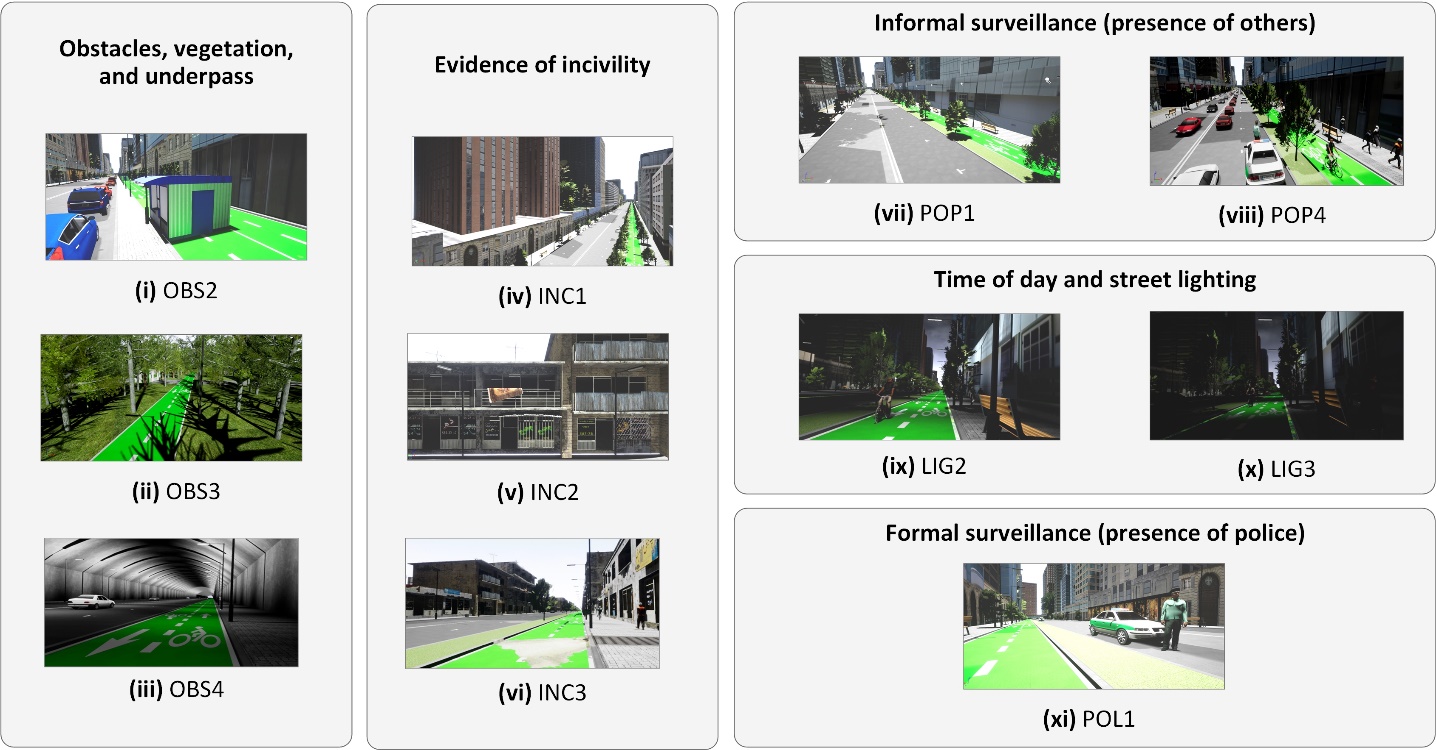


**Supplementary Figure S1.** Variety of settings of factors affecting the perception of security, designed in the virtual reality environment.

. . . . . . . . . . . . . . . . . . . . . . . . . . . . . . . . . . . . . . . . . . . . . . . . . . . . . . . . . . . . . . . . . . . . . . . . . . . . . . . . . . . . . . . . . . . . . . . . .


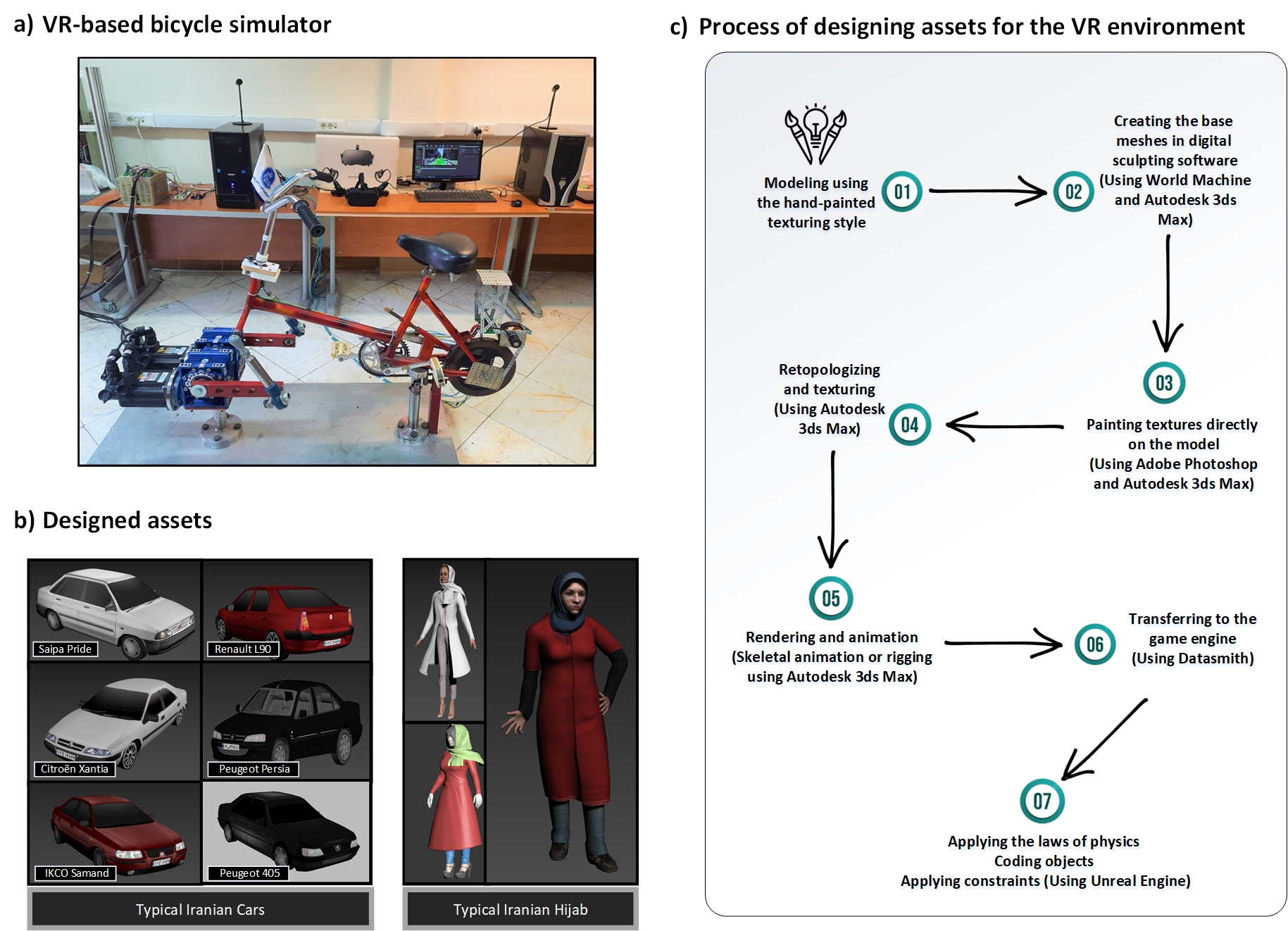


**Supplementary Figure S2.** VR-based bicycle simulator.

. . . . . . . . . . . . . . . . . . . . . . . . . . . . . . . . . . . . . . . . . . . . . . . . . . . . . . . . . . . . . . . . . . . . . . . . . . . . . . . . . . . . . . . . . . . . . . . . .


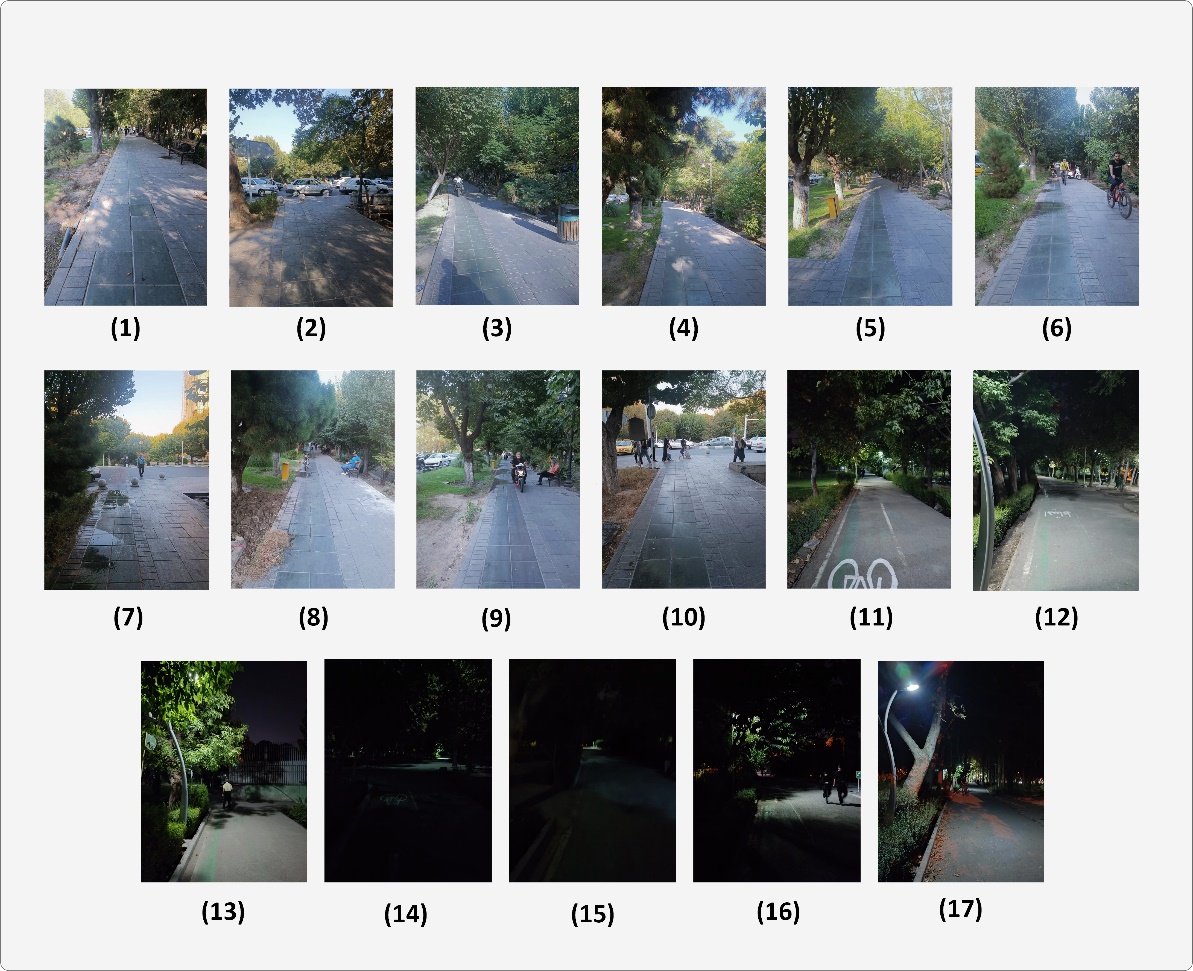


**Supplementary Figure S3.** Real view of Laleh Park and Keshavarz Boulevard stations in Tehran.

. . . . . . . . . . . . . . . . . . . . . . . . . . . . . . . . . . . . . . . . . . . . . . . . . . . . . . . . . . . . . . . . . . . . . . . . . . . . . . . . . . . . . . . . . . . . . . . . .
